# Artificial Intelligence and Internet of Things Integration in Pharmaceutical Manufacturing: A Smart Synergy

**DOI:** 10.3390/pharmaceutics17030290

**Published:** 2025-02-22

**Authors:** Reshma Kodumuru, Soumavo Sarkar, Varun Parepally, Jignesh Chandarana

**Affiliations:** 1 KBI Biopharma, Inc., Durham, NC 27704, USA; 2 Novartis AG, East Hanover, NJ 07936, USA; ss3627@cornell.edu; 3 Chemical Engineering Department, Michigan Technological University, Houghton, MI 49931, USA; varunparepally@gmail.com; 4 Grail Inc., Menlo Park, CA 94025, USA; onlyjignesh@gmail.com

**Keywords:** artificial intelligence (AI), drug discovery, machine learning, real-time monitoring, internet of things (IOTs)

## Abstract

**Background:** The integration of artificial intelligence (AI) with the internet of things (IoTs) represents a significant advancement in pharmaceutical manufacturing and effectively bridges the gap between digital and physical worlds. With AI algorithms integrated into IoTs sensors, there is an improvement in the production process and quality control for better overall efficiency. This integration facilitates enabling machine learning and deep learning for real-time analysis, predictive maintenance, and automation—continuously monitoring key manufacturing parameters. **Objective:** This paper reviews the current applications and potential impacts of integrating AI and the IoTs in concert with key enabling technologies like cloud computing and data analytics, within the pharmaceutical sector. **Results:** Applications discussed herein focus on industrial predictive analytics and quality, underpinned by case studies showing improvements in product quality and reductions in downtime. Yet, many challenges remain, including data integration and the ethical implications of AI-driven decisions, and most of all, regulatory compliance. This review also discusses recent trends, such as AI in drug discovery and blockchain for data traceability, with the intent to outline the future of autonomous pharmaceutical manufacturing. **Conclusions:** In the end, this review points to basic frameworks and applications that illustrate ways to overcome existing barriers to production with increased efficiency, personalization, and sustainability.

## 1. Introduction

Fast-growing consumer needs, along with a rapid growth in technology, have implored the industries present in this modern arena to adopt new methodologies that can help them in enhancing their operational efficiencies relating to quality improvement [1]. Amongst all such industries, pharmaceuticals hold a considerable position in health sectors and hence are keen to investigate new ways for process improvement and product upgrading [2]. With these growing global health emergencies, coupled with the high demand from the COVID-19 pandemic, this has accelerated the call to apply more innovative technologies in different operations to more easily achieve better outcomes [3]. The pandemic has acted as a strong catalyst for change within the pharmaceutical industry by pointing out weaknesses in many of the traditional systems and fast-tracking the need to optimize manufacturing, drug development, and distribution networks [4]. Thus, it has shown the way to integrate artificial intelligence and the internet of things into the improvement of efficiency and productivity in pharmaceutical manufacturing [5].

**Figure 1:**
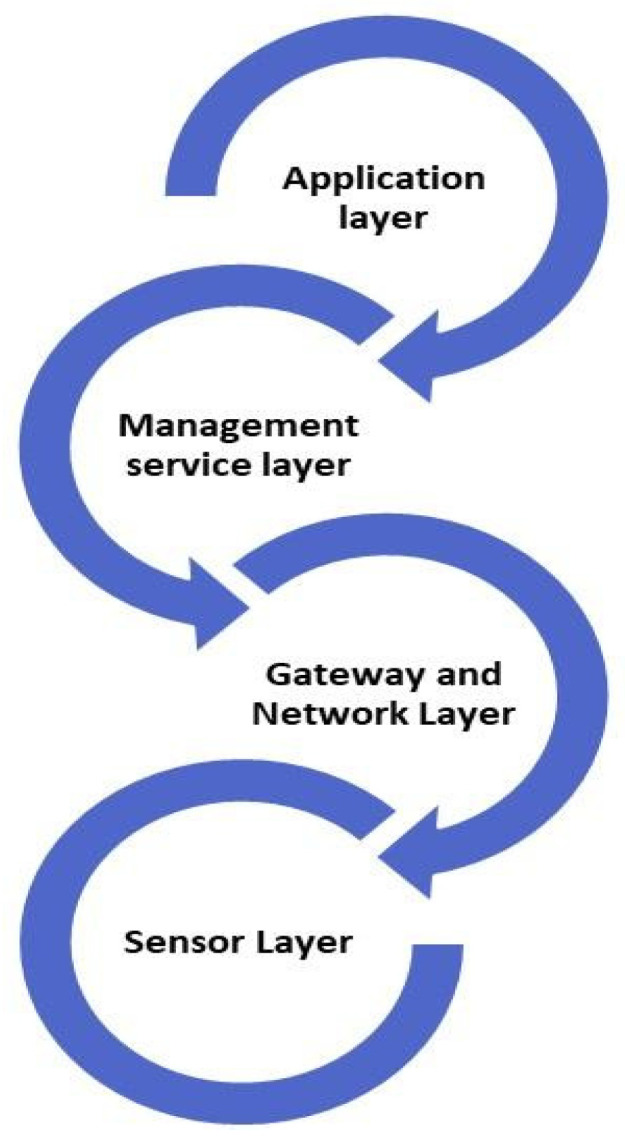
The architecture of the IoTs.

AI and the IoTs have started redefining the face of the pharmaceutical landscape and have, therefore, become transformational technologies [6]. AI consists of all those broad-ranging technologies, from machine learning to natural language processing, among other things, to afford advanced analytics with automation capabilities (Table 1). Similarly, the IoTs connects various diverse devices and sensors, as Figure 1 shows, thereby connecting data exchange with real-time monitoring without the involvement of any device or manual interference [7]. Put together, these technologies can enable a quantum leap in operational effectiveness, quality of products, and regulatory compliance from the conventional, inefficient manual process followed in the industry [8].

Traditionally, the manufacture of pharmaceuticals has been highly labor-intensive, with a heavy reliance on human intervention and resulting errors [9]. QA is often confined to the sphere of being merely reactive at its best and very expensive regarding recalls and other resource waste [10]. In addition, it is precisely the traditional regulatory framework that brings in a lot of paperwork, standing in the way of agility and responsiveness. On the other hand, it could be that with the downline integration of IoTs sensors in a line of production (Appendix B), the capacity for continuous monitoring of shifts in critical parameters moves away from mere end-of-line testing to proactive quality assurance, whereby immediate action can be taken against any deviations in quality to reduce waste and ensure compliance [11].

AI further seals this change through the implementation of machine learning algorithms analyzing enormous datasets in search of trends that predict near failures [12]. In other words, that is precisely what predictive maintenance is, other than the normal trend where machines would often be scheduled for service on random timelines without regard to actual performance [13]. This can help pharmaceuticals reduce unplanned downtimes, whilst also improving overall effectiveness through the optimization of various production parameters for better yield and consistency in the quality of the end products [14].

Although AI and the IoTs contribute a lot with their benefits, it is not that easy to transition from conventional manufacturing. High costs at the initial stage and the complexities involved in integrating new technologies pose serious obstacles, especially to SMEs [15]. The automation of certain roles within the workforce also raises the need for the upskilling of employees amidst a backdrop of new responsibilities that will involve these advanced technologies [16].

**Figure 2:**
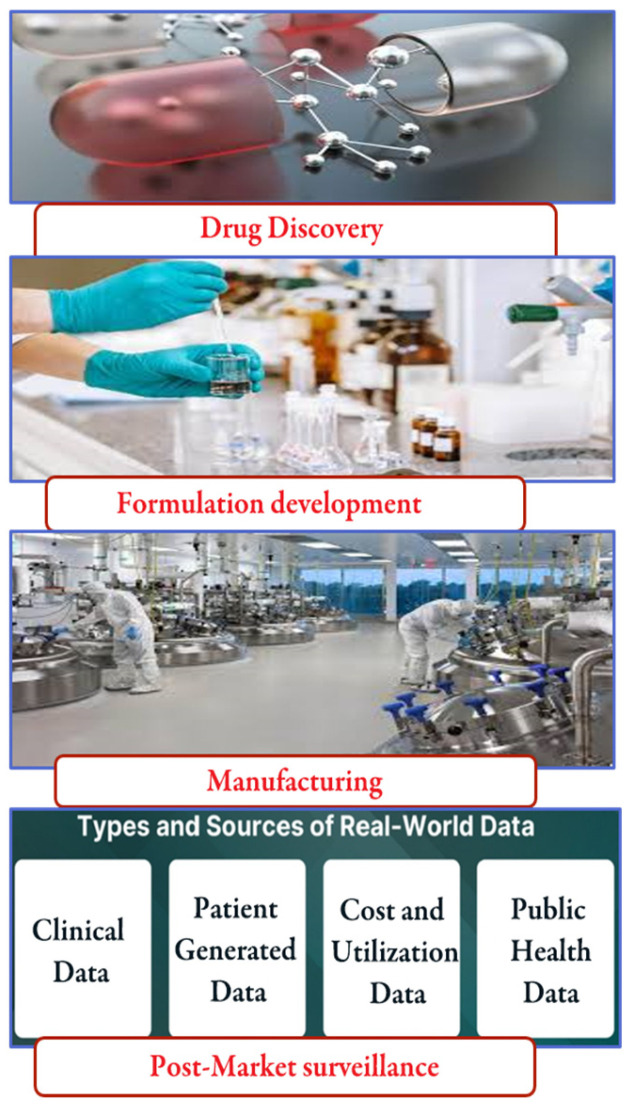
Application of artificial intelligence in enhancing the drug development and distribution life cycle.

Effective ethics and regulatory frameworks would have positioned the inclusion of AI and IoT in pharmaceutical manufacturing as a last resort, as illustrated in (Figure 2) [17]. Many concerns have been raised over integrity, algorithmic thinking with bias, and personal data amongst others due to the sensitivity of the information that organizations collect [15]. This is where all regulators and industry leaders should have come together to make sure everything was to the set standards. Consequently, the regulatory frameworks must respond to the respective changes in technology while promoting innovation and protecting public health [11].

### Objectives of the Review

The aim of this review is the presentation of the transformational impact brought in by the integration of AI and the IoTs in the pharmaceutical manufacturing industry. We try to answer a few relevant questions that are listed as follows:

– Which technological frameworks enable the integration of AI and the IoTs?
– How will these technologies enhance operational efficiency along with compliance?
– What are the implementation challenges related to organizations, and how to overcome them?
– What are the implications of AI and the IoTs on workforce dynamics? How can the employees be upskilled towards adapting to new emerging roles?

This review will therefore answer these questions and give an appropriate understanding of the actual state of the art in the integration of AI and the IoTs in pharmaceutical manufacturing, hence outlining possible pathways for future research and implementation.

This is a conceptual leap from anything that has prevailed so far: the integration of AI and the IoTs into the manufacturing process. These various solutions ensure quality and productivity improvement, as well as meeting regulatory compliance by way of real-time monitoring, predictive maintenance, and data-driven decision-making. The implementation of the model needs to be performed after overcoming the challenges that come along the way so that the overall benefit can actually be achieved in integration, without which a well-balanced approach toward innovation shall always be uncompromising with safety and protection of citizens’ health.

Clinical data contain electronic health records and case report forms. Patient-generated data contain health and treatment history, biometric data, and patient-reported outcomes. Cost and utilization data contain claims datasets, biometric data, and public databases; public health data contains government data and national networks.

## 2. Overview of AI and the IoTs in Industrial Applications

Artificial intelligence and the internet of things have been two disruptive technologies with major applications in modern times, for which their origins were considerably far-reaching. While these are cutting-edge applications now, their development came rather successively, stage by stage, transforming them into what is present nowadays [18,19].

### 2.1. Evolution and Growth of the AI and IoTs Technologies

The concept of the IoTs started in the early 1980s when some researchers at Carnegie Mellon University connected a Coca-Cola machine to the internet so its stock and functionality could be kept in check. This was to be the first step toward networking physical objects. In 1999, Kevin Ashton named it the “Internet of Things” and described a scenario in which all objects would communicate with each other over the Internet [20,21].

In the 2000s, the IoTs entered a phase of rapid expansion due to increased wireless networking, sensor technologies, and cloud computing. Since the 2010s, the IoTs has been applied everywhere, from smart homes to industrial automation. Such systems can gather a huge volume of real-time information that industries can depend on for optimization and improving efficiency [22,23].

### 2.2. Technological Underpinning of AI and IoTs Integration

In fact, some key technologies rooted in communications, data processing, and real-time analytics can effectively integrate with AI and the IoTs [24]. According to Table 2, it involves some of the most important ones like machine learning algorithms, computer vision, robotics and automation, cloud computing, edge computing, digital twin technology, cybersecurity, and data management systems.

#### 2.2.1. Key Technologies Enabling Integration Between AI and the IoTs

##### Algorithms for Machine Learning and Deep Learning

In recent years, the role of machine learning and deep learning algorithms for enhancing the potential of any AI-driven system in dealing with huge volumes of data, as provided by IoTs devices, has increased many folds [25,26]. Such algorithms thereby facilitate self-learning to make autonomous executions for predictions, optimizations, and decision-making. Supervised learning often finds applications in data that are labeled, whereas unsupervised learning contributes towards pattern recognition over unlabeled data for anomaly detection and real-time decision-making in a system, as proposed by [27,28]. On the other hand, reinforcement learning offers continuous improvement through a feedback mechanism that has many uses in dynamic environments.

While all these developments sound so exciting in machine learning, the unease grows due to the ethics behind those technologies. This would only be one example of the need for more transparency within the decision-making process of AI; these are some of the questions that will be continuously discussed. It is somehow necessary for the future that ethical reflection in relation to development and deployment is a constitutive element when it comes to building trust and fairness in automated systems.

##### IoTs Frameworks and Communication Protocols

The underlying effective communication protocols are essentially about the ability of IoTs devices to share information freely with AI systems. Different protocols have been employed in different operational environments [29,30]. A middleware platform helps in orchestrating the interactions between the devices and the AI infrastructure [31].

From diverse perspectives, researchers proved that lightweight protocols were very efficient under constrained conditions, but the scalability issue of such protocols in large systems was still a concern [32,33]. IoTs network performance is greatly dependent upon the right choices of protocols, hence such factors should be among prime consideration at the time of designing the system. Therefore, the choice of protocol is required to be flexible or adaptive in nature if implementation in diverse applications is a concern for optimum performance.

##### Cloud and Edge Computing

IoTs devices generate data volume with exponential growth, and huge computation resources are needed which are effectively managed by cloud and edge computing. Major cloud platforms, including AWS, Google Cloud, and Microsoft Azure, have come up with scalable infrastructure to handle IoTs data processing and analytics. Thus these enable facilitation in remote data monitoring, storage, and the deployment of machine learning models. In contrast, edge computing reduces latency by processing the data near its source—either directly on IoTs devices or on edge servers—and then sending it to the cloud. This capability is particularly crucial for those applications that require real-time processing, such as autonomous vehicles and industrial automation [34,35].

As much as cloud computing has become integral, it is also a big concern regarding data privacy and security, since data can be transferred and stored even from other locations. Secondly, there is unequal dissemination because of the differences in infrastructure between the developed and developing parts of the world. However, challenges need to be overcome with secure and efficient cloud solutions for better security of data and wider access.

##### IoTs Sensors and Devices

IoTs sensors are core in capturing data to enable AI systems to make decisions based on that data [36]. Temperature and humidity sensors are just a few examples of sensor types deployed in different industries, such as the pharmaceutical one. Motion and vibration sensors support predictive maintenance by monitoring irregular machinery behavior.

While exciting times are abreast regarding the use of IoTs sensor technology, there has to be rigorous safety and regulatory standards for such applications, at least for sensitive vertical industries such as pharmaceuticals. Again, it involves proper coordination among the players, regulators, and providers to arrive at best practices in order to make sure there is responsible deployment of IoTs technologies.

##### Data Management and Analytics

Data management itself plays a very important role in the integration of AI with the IoTs as these IoTs emitting devices will give off a tremendous amount of data that need to be updated, stored, and analyzed, and on which the AI algorithms (Appendix A) act for insight generation to drive automation [37].

A. Data Collection and Preprocessing: Before any AI model can begin processing IoTs data, these data must be first collected, cleaned, and preprocessed. Aggregating platforms like Apache Kafka and Apache NiFi help in gathering data from several IoTs devices and putting it together for further analysis. Some of the necessary data preprocessing techniques for quality and for making it suitable for training AI models include normalization, cleaning of data, and feature extraction [38].

B. Data Storage and Big Data Management: Conventional databases will not be effective for storing the volume of real-time data emanating from IoTs devices. Examples of big data storage include MongoDB, Cassandra, and other types of NoSQL databases along with distributed file systems like Hadoop HDFS. These systems can store large amounts of data coming from sensors and fetch the data for efficient processing by an AI model [39,40,41].

C. Data Analytics and Processing Platforms: Once the information is stored, the information then needs to be processed and analyzed so that meaningful insights can be extracted from it. Apache Spark and TensorFlow are some popular tools which process huge amounts of data, do real-time analytics, and apply machine learning algorithms on them. Quite a few AI models have been developed on these platforms with the purpose of outcome prediction, anomaly detection, and process optimization in industries [36].

D. Real-Time Data Streaming: Most IoTs applications are based on real-time data processing. Some of the widely used platforms that stream data from IoTs devices to immediately act on analytics platforms by AI models include Apache Kafka, Apache Flink, and AWS Kinesis. This is very important in applications like predictive maintenance and dynamic manufacturing optimization where immediate action needs to be taken on the incoming data [42,43,44].

The integration of AI and the IoTs holds immense potential to change many more industries than the pharmaceutical one, including manufacturing, energy, transportation, and agriculture. The integration of various machine learning algorithms, the framework of the IoTs, cloud and edge computing, and specialty sensors will, in turn, enable AI-IoTs systems to realize real-time decision-making, optimization, and automation. Since these are still continuously evolving technologies, their shared impact on industrial processes has been one of increasing innovation, sustainability, and efficiency enough to change the face of industrial operations across industries in general [45,46,47].

#### 2.2.2. Future Directions and Call to Action

Coupled IoTs and AI hold immense promise for other industries than the ones mentioned, including pharmaceutical manufacturing, energy, transportation, and agriculture [48]. Since the technologies themselves keep moving further in innovation, their influence on the innovation and efficiency of industrial processes will also ensure the principle of sustainability is adhered to, as was reflected by [49,50].

However, important domains indeed need some fundamental further research and development in, such as the following:

– Ethical AI: This is about creating a framework of transparency, accountability, and fairness in AI decision-making so that the stakeholders can develop a certain sense of trust. Indeed, much more research is still being carried out on how to implement ethical performance in AI, reducing bias and presenting results equitably [51,52].
– Security and Privacy: Security at both the cloud and edge should be enhanced to keep this type of sensitive data protected. Advanced techniques of encryption, use of secure protocols in the process of transferring the data, and strong authentication reduce the risk [53].
– Scalability of Protocols: Where warranted, a search shall be engaged for scalable communication protocols that are capable of handling large-scale IoTs networks. In addition, the development of protocols considering operational environments in all dimensions would enable wide-scale adoption of AI-IoTs solutions [54].
– Global Regulatory Standards: There is a need for a common standard regarding IoTs sensors, data privacy, and security so that more responsibilities could be granted toward IoTs deployment across different industrial and geographical boundaries [55].

#### 2.2.3. Step-by-Step Process for Integrating AI and the IoTs

Several industries strive toward the integration of AI and the internet of things into a comprehensive systematic process, hence requiring the inclusion of their respective challenges [56]. The concrete steps toward the aforementioned goals are underlined hereafter:

– Pilot Programs: These are small pilot projects to test the effectiveness of AI-IoTs integration for certain use cases. The pilots thus allow for controlled testing, whereby organizations can identify potential challenges and assess the benefits that come with integration before full-scale implementation [57].
– Infrastructure Assessment: Assessing the current infrastructure for necessary upgrades or replacements. This assessment of compatibility with the selected AI and IoTs technologies ensures a smooth integration process [58].
– Selection of Communication Protocols: The selection of the protocols shall be made in view of operational requirements, data types, and scalability needs. Indeed, protocol selection is very crucial concerning performance optimization for reliable data exchange [59].
– Data Management Strategy: An elaborative proper data management strategy will be provided, focusing on how the information will be gathered, stored, preprocessed, and analyzed. The strategy should have the capability for volume and speed from IoTs gadgets and good integrations with AI algorithms.
– Training and Development: Impart training programs to the personnel to acquire the necessary skills to work with new technologies. The employees must be well versed in AI and IoTs systems for smooth implementation.
– Implementation: The integrated system is to be implemented incrementally, first with the components that worked well during the piloting of the program. The period will require a constant follow-up and evaluation process to be performed, solving emerging problems during the process.
– Loops for Feedback: A design methodology by which the users and the systems will feed information continuously on an improvement basis. To this end, an organization will have a fine-tuned AI-IoTs system by having periodic collections of feedback or analysis for improvement of performance.
– Scale Up: Scaling up integration based on the success of the pilot programs and initial implementation in various departments or applications. Performance assessment from time to time, with necessary tuning, also constitutes the scaling-up processes.

#### 2.2.4. Common Challenges at Implementation and How to Override

AI and the IoTs face challenges in an integrated environment. To make the integration successful, organizations should be proactive regarding potential obstacles in its way, including:

– Data Security: A very secure system with encryption protocols needs to be established to ensure sensitive data are safe throughout the integration process. Regular security audits are to be performed to find vulnerabilities to minimize risks [60].
– Interoperability: Standards-compliant devices and systems shall be selected to offer compatibility on different platforms. In fact, interoperability is at the core of any effective communication and data exchange within an integrated system [61].
– Change Management: Organizational buy-in shall be ensured through explicit articulation of benefits and impacts due to AI-IoTs integration. The involvement of stakeholders and resolution of their concerns will yield a supportive implementation environment [62].
– Continuous Improvement: An enabling environment for continuous learning through experimentation and lessons learned, in case of failure, calls for the ability of an organization to adapt and iterate approaches as it learns [63,64].

If these steps are followed in a structured manner and any challenges which arise are resolved, then industries can successfully integrate AI and the IoTs, unlocking huge benefits and driving transformative changes across industries. Since the technology landscape is changing continuously, no doubt the collaborative potential of AI and the IoTs will shape the future of industrial operations: more efficient, greener, and more innovative [65].

**Table 2:** Summary of IoTs and AI technologies considered in enhancing pharmaceutical manufacturing.

Technology	Category	Description	Applications in Pharmaceutical Manufacturing	Reference
Machine Learning (ML)	AI technology	Algorithms that enable systems to learn from data and make predictions without explicit programming.	– Predictive maintenance of production equipment. – Demand forecasting for production planning. – Quality control and anomaly detection.	[59]
Computer Vision	AI technology	An AI subfield that trains systems to interpret and understand visual information, such as images or video.	– Automated inspection of tablets, packaging, and labeling. – Monitoring production lines for quality assurance. – Detecting defects in packaging.	[60]
Robotics and Automation	AI and IoT technology	Use of robots and automated systems to perform tasks traditionally requiring human intervention, improving efficiency and consistency.	– Automated pharmaceutical production lines. – Robotic systems for packaging, labeling, and sorting drugs. – Handling hazardous chemicals or materials.	[61]
Cloud/Fog/Edge Computing	IoTs and AI technology	Architectures that allow data processing to be performed in a distributed manner—either on the cloud, fog, or in edge devices—enabling real-time decision-making and reducing latency.	– Real-time monitoring of production conditions. – Cloud-based data storage for research and manufacturing data. – Edge computing for real-time control.	[62]
Digital Twin	IoTs and AI technology	Virtual replicas of physical objects or systems used to simulate, monitor, and optimize the performance of physical assets.	– Simulating and optimizing drug production processes. – Predicting the performance of manufacturing equipment. – Testing new formulations virtually.	[63]
Cybersecurity	AI and IoT technology	Technologies aimed at securing data and protecting systems from unauthorized access, breaches, or attacks.	– Protecting patient and drug manufacturing data. – Ensuring regulatory compliance and data integrity. – Preventing cyber threats in connected devices.	[64]
Data Management Systems	IoTs and AI technology	Platforms and systems that handle the collection, storage, analysis, and management of large volumes of data generated by IoTs devices and AI systems.	– Storing and processing pharmaceutical manufacturing data. – Enabling data-driven decision-making. – Supporting regulatory reporting and compliance.	[65]

## 3. Case Studies of AI and the IoTs in Drug Manufacturing

**Figure 3:**
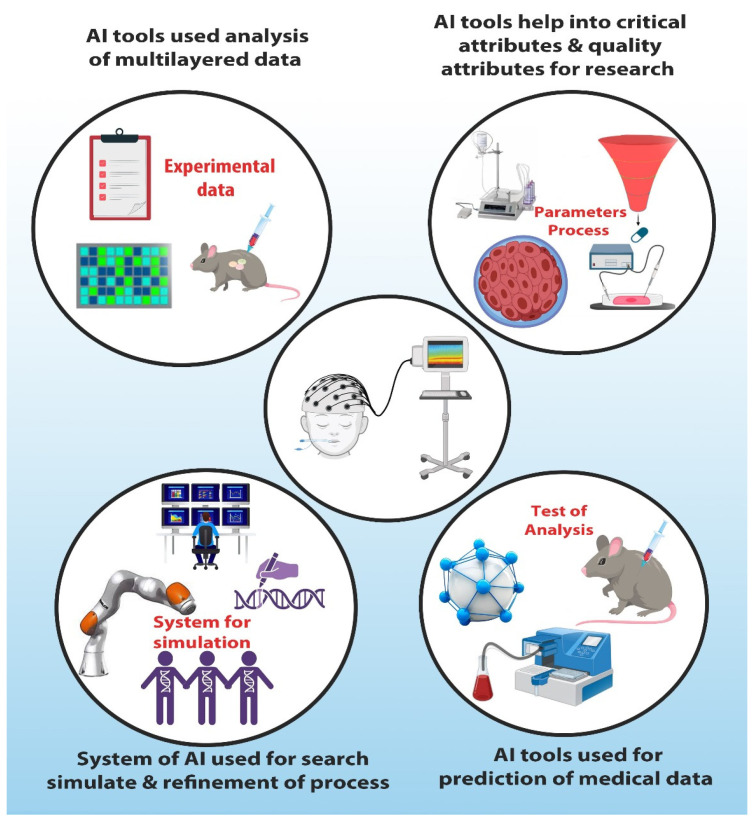
Application of AI tools in the pharmaceutical sector.

Figure 3 illustrates the paradigm shift toward new levels of operational efficiency, quality control, and personalization of medical treatments by integrating AI and the IoTs into pharmaceutical manufacturing. Of these, some of the key case studies reviewed demonstrate not only successful implementations but also intrinsic challenges and wider ramifications of these technologies within the pharmaceutical landscape, as discussed in Table 3.

AI-driven predictive maintenance at Novartis is a strategic response to the persistent issue of equipment downtime that characterizes drug manufacturing. Novartis uses IoTs sensors that continuously monitor the condition of critical equipment in real-time, feeding data into AI algorithms that predict potential failures [66,67]. This approach has led to substantial decreases in unplanned downtime and increased equipment effectiveness overall. Predictive models have to be reliable, though, and predictive models are only as good as the quality and accuracy of the sensor data collected. Poor-quality data can trigger false positives or negatives in failure predictions that may disrupt the production line rather than improve it. In addition, AI and the IoTs integrated with legacy systems remain a big challenge because many manufacturing facilities operate on older technologies that do not mesh well with newer solutions [68].

Real-time monitoring of tablet production at AstraZeneca is yet another case at the critical juncture between technology and regulation [2]. IoTs sensors monitor weight and hardness to maintain high standards of medicines with minimal generation of rejects and human errors. However, these systems also have their own shortcomings, such as sensor calibration and the accuracy of the data. Such anomalies in sensor readings may lead to faulty process adjustments with serious consequences on product quality and patient safety. It also creates an overreliance on automation that abstracts operators from the niceties of quality control and potentially diminishes situational awareness [69,70].

Applications of the IoTs for environmental monitoring in sterile manufacturing at Eli Lilly pin the prime environment for contamination prevention against the production of injectables. As exemplified by [1], air quality, humidity, and temperature continuous tracking are performed through IoTs-enabled sensors and thus there is real-time input. Eli Lilly can then act very fast on deviations. In contrast, there is a huge need for fitting several sensors. Advanced AI analytics is required to manage such data and infer from it efficiently. If not supported by some sound strategy or well managed, timely decisions may also be influenced negatively by information overload. Apart from that, it is questionable if these monitoring systems could be exposed to cyber-attacks and what would be the options in case something goes wrong with them [70,71].

Artificial intelligence is used at Roche for personalized formulation of drugs: This can be termed the ultimate advance toward personalized medicine, the tailoring of therapies to the particular profile [72,73]. However, scalability is still an issue in most instances because much of the manufacturing infrastructure currently is designed for bulk production. These will need to be revision in light of personalized therapies, a process that requires huge investment and a cultural shift in organizations aligned largely with traditional operations. Further, ethical and regulatory challenges—most of them with sensitive patient data—are at the forefront, especially under stringent regulations such as HIPAA in the United States. Robust cybersecurity measures have to be put in place to guard the data to ensure compliance and ethical use [74].

AI-powered automated packaging systems at Pfizer represent another innovation aimed at enhancing safety and compliance in drug manufacturing. Pfizer’s AI-driven vision inspection systems significantly reduce packaging errors while increasing throughput [75,76]. However, these systems face a critical limitation: extensive training of AI models is required to identify even minor defects. Yet, undertraining may cause defects that might not be revealed and may even lead to patient safety threats. Thirdly, AI systems are also described as complex and time-consuming to integrate, with existing workloads of packaging a trend so general in the industry: great benefits from AI and the IoTs should be weighed against operational obstacles [77].

### 3.1. Importance of Data Integrity

Importance to Pharmaceutical Manufacturing: The integrity of the data themselves allows for the derivation of accurate models and automation systems that develop reliable predictive performance in pharmaceutical applications [78]. Precise, complete, and reliable data at all instances–from creation to the whole life cycle–is essential to derive proper decisions. Poor data integrity will lead to bad predictions, poor-quality products, and violations in regulations affecting patient safety [79,80].

Apache Kafka will be used for real-time data streaming, Apache NiFi for ingestion and processing, while a NoSQL database, such as MongoDB, can be chosen for data storage as it scales well [81,82]. These will enhance data integrity since there will be appropriate frameworks to validate data, process them, and store the same securely [83].

### 3.2. Cybersecurity Threats and Measures

However, increased integration of AI and the IoTs into pharmaceutical manufacturing exposes companies to cybersecurity threats like data breaches, ransomware attacks, and unauthorized access, which, when successful, result in operational disruption, compromise sensitive patient data, and eventually lead to regulatory non-compliance [84].

The companies are expected to adopt recent security measures to reduce this risk to the bare minimum. This should be in the form of the following [85]:

– Encryption: it provides protection against unauthorized access to data both at rest and in transit.
– Access Controls: the implementation of RBAC can help reduce the access to data by unauthorized people and insider threats.
– Regular Audits: regular security audits and vulnerability assessments identify the potential weaknesses of the system.
– Incident Response Plans: formulating comprehensive incident response plans helps organizations to respond quickly and effectively during cybersecurity incidents.

### 3.3. Case Studies Outcome Evaluation

The case studies presented herein regarding the integration of AI with the IoTs in pharmaceutical manufacturing have brought both successes and challenges [86]. Though predictive maintenance at Novartis significantly reduced downtime, the dependency on data quality raises the need for stringent data management protocols. Similarly, real-time monitoring greatly improved the quality control of AstraZeneca; however, sensor accuracy and automation vulnerabilities do put patients’ safety at risk [87,88].

On the contrary, at Eli Lilly, environmental monitoring underlines the need for advanced analytics, but data overload and cyber threats outnumber them. Personalized medicine development by Roche reflects the opportunities of AI in transformation but at the same time shows the investment needs regarding infrastructure and ethics [89]. Automation of packing at Pfizer illustrates the possible operational efficiencies arising from AI, while the risk of missed defects underlines that thorough integration plans and, particularly, training will be needed [90].

In other words, while promising much for pharmaceutical manufacturing in terms of better efficiency and quality, AI and the IoTs face different challenges: data integrity, system integration, scalability, and ethical data management [91].

### 3.4. Challenges and Case Examples in Compliances

Many challenges arise while deploying AI and the IoTs technologies in order to meet the set regulations, especially data privacy and quality standards that pharmaceutical firms have to pay full heed to [91]. In other words, good, automated manufacturing practice calls for a degree of challenge in its setup. It has to be balanced with the quest for innovation, seeking balance with compliance obligations in complex regulatory systems [92].

Companies like Eli Lilly have overcome these kinds of challenges by developing a holistic strategy for real-time environmental monitoring within the tightest GMP guidelines [93,94]. Hefty investments in advanced analytics and cybersecurity enable Eli Lilly to stand up to certain regulatory scrutiny [95].

The proactive stance taken by AstraZeneca in implementing real-time monitoring underlines that transparency regarding quality maintenance and automated systems, complemented by human oversight, is indeed very important. Since quality control is embedded within the automated processes, AstraZeneca can reduce the risk of inaccuracies with sensors and potential regulatory non-compliance [96,97].

In a nutshell, the application of AI and the IoTs in pharmaceutical product manufacturing provides several realistic opportunities for many challenges that are overwhelming [98]. In addressing data integrity, evaluating and mitigating threats to cybersecurity, making considerations on the case outcome, and having some knowledge on how compliance matters are tackled, pharmaceutical organizations shall place themselves in positions to take full advantage of such technologies to assure patients of their safety as well as the fulfillment of regulatory necessities [99].

Future innovation in this sector must balance operational efficiency with the core tenets of patient safety and compliance [100,101]. A dialog between emerging technologies and traditional manufacturing practices will continue to shape the future of drug manufacturing, emphasizing responsible advancements that ensure the integrity of the production process and the well-being of patients.

**Table 3:** List of companies using AI and ML technologies in pharmaceutical research as discussed by [102,103,104].

No.	Domain	Technology and Outcome	Industry Collaborations
**1**	Drug Design	Development of novel therapeutic antibodies	Exscientia
**2**	Molecular Discovery	Deep learning platform for structure-based drug design	AtomWise
**3**	Genetic Research	Machine learning for gene mutation analysis	Recursion
**4**	Drug Design	Ligand- and structure-based de novo drug design	Iktos
**5**	Drug Discovery	Generative modeling for drug discovery	Iktos and Galapagos
**6**	Drug Development	Identification of preclinical candidates	Iktos and Ono Pharma
**7**	Rapid Drug Design	Software tool “Makya™”, (https://iktos.ai/solution/makya; accessed date: 21 February 2025) for accelerated drug design	Iktos and Sygnature Discovery
**8**	Comprehensive Discovery	Pharma.AI and related platforms for drug discovery	Insilico Medicine
**9**	Drug Targeting	AI-based tool for drug target identification	Insilico Medicine
**10**	Drug Development	Analyzing protein dynamics in drug development	Relay Therapeutics
**11**	Drug Discovery	AI-driven selection for drug target identification	BenevolentAI
**12**	Target Discovery	Drug target selection for chronic diseases	BenevolentAI with AstraZeneca, GSK, and Pfizer
**13**	Clinical Trials	AI integration for optimizing clinical trials	Pfizer and Vysioneer
**14**	Disease Treatment	AI and supercomputing for COVID-19 treatment	Pfizer
**15**	Drug Discovery	Novel NASH therapies using AI	AstraZeneca and Viking Therapeutics
**16**	Clinical Trials	AI platform for clinical trial site feasibility and recruitment	Janssen
**17**	Research Automation	Automated literature review using NLP	Sanofi
**18**	Drug Development	AI-driven approaches in drug development	BioMed X and Sanofi
**19**	Research Collaboration	AI exploration platforms for drug research	Novartis and Microsoft
**20**	Drug Discovery	AI platform for drug discovery	Bayer

## 4. Paper Research Methodology

### 4.1. Literature Review and Selection Criteria

A literature search was systematically conducted to provide an extended review of how AI and the IoTs are applied in pharmaceutical manufacturing, using several university databases: PubMed, Scopus, IEEE Xplore, Springer, and Web of Science, to identify pertinent articles focusing on the integration of the IoTs and AI in pharmaceutical manufacturing. Article selection was based on predefined eligibility inclusion and exclusion criteria by analyzing titles, abstracts, and keywords.

#### Inclusion and Exclusion Criteria

A. Inclusion Criteria.
  – Peer-reviewed English articles discussing the integration of the IoTs and AI in pharmaceutical manufacturing.
  – Full-text articles.
  – Articles published from 2019 to 2023.
  – Empirical research, case studies, and systematic reviews.
B. Exclusion Criteria.
  – Any articles that are not peer-reviewed ones.
  – Articles with abstracts only and not full-text articles.
  – Articles published in any language other than English.
  – Articles published outside the duration of 2019 to 2023.

This review ranges from 2019 to 2023 because there is concentrated research within efforts during that period. The search was for English journal articles that were peer-reviewed and in the fields of the internet of things and artificial intelligence applied to pharmaceutical manufacturing. Inclusive materials included empirical research, case studies, and systematic reviews that claimed to state an outcome measuring their efficiency in cost reductions, enhancement of quality, or adherence to regulatory compliances. At this stage, various views and results relevant to the identified problem statement could be compiled. The problem statement was the first building block, clearly and effectively framing several challenges and opportunities that the integration of AI and the IoTs presents for pharmaceutical manufacturing. This entailed bringing the focus onto only those problems that actually needed investigation and thus outlined the ground wherein meaningful investigation could occur.

**Figure 4:**
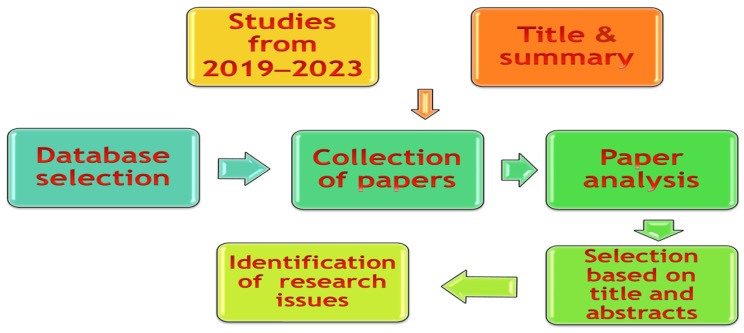
Research methodology.

In all, a total of 300 articles were identified within the databases. An actual analysis of the abstracts was carried out to exclude works not relevant to the research. The documentation search process is schematically presented in Figure 4. Keywords used in the search are as follows: “Artificial Intelligence in pharmaceutical manufacturing, IoTs applications in drug production, integration of AI and IoTs, Predictive maintenance in pharmaceuticals, and real-time quality monitoring”. This was a deliberate move to ensure that the studies referred to are recent works, showing the most current advancement in the field and reflecting the ever-changing technological face of the pharmaceutical industry. However, seminal works which established foundational theories were also included to provide a historical perspective. After removing the duplicates, 115 peer-reviewed articles were selected for the final review to enhance credibility and scholarly rigor.

While the literature review has shed enough light on integrating AI and the IoTs in pharmaceutical manufacturing from 2019 to 2023, there are still gray areas that need further review

### 4.2. Areas Needing Further Research

The following areas merit additional research:

– Long-term Sustainability and Scalability: It will be interesting to see how these various deployments of AI and the IoTs will actually be effectively sustainable and scaled into the various pharmaceutical manufacturing sites. Hence, this study will include best practices and frameworks for sustaining these operations long term with minimal operational challenges in the way of resource allocation and technology upgrades.
– Interoperability with IoTs devices, considering AI systems with legacy infrastructure, is complex; hence, interoperability research between both remains an area that needs further study. Standard and protocol development that enables easy communication among diverse technologies could facilitate better integrations.
– Bias and Accountability in Decision-making by AI Algorithms: Above all, from the point of view of ethics, more ground is expected to be considered with more stringency. Moreover, the ever-evolving regulatory landscape really warrants a study that would analyze implications for compliance with AI and the IoTs in light of emerging technologies.
– Security and Privacy of Data: With such rapid integration, there are a number of emerging data that raise very serious concerns, and hence research should be targeted toward strong cybersecurity to protect sensitive information. That is, a place for the inclusion of data governance frameworks concerning privacy issues, considering compliance with prevailing laws such as the General Data Protection Regulation.
– Workforce Dynamics: The impact of AI and the IoTs adoption on workforce dynamics will have to be understood. Research is needed into how these technologies are affecting job roles, required skills, and training needs in pharmaceutical manufacturing, and strategies for workforce adaptation.
– Case Studies of Successful Implementation: More empirical case studies highlighting specific success factors and lessons learned from organizations that have so far integrated AI and the IoTs into their operations would be instructive. Therefore, these would provide practical insights to guide other firms in their journeys of adoption.
– Quantitative Metrics for Impact Measurement: Standardized quantitative metrics should be laid down to measure the impact of AI and the IoTs on the key performance indicators related to pharmaceutical manufacturing. This research shall aim at constructing conceptual frameworks that could support consistent evaluation of advantages in quality, efficiency, and compliance.
– Consumer Perspective and Supply Chain Integration: Future research should be performed considering the consumer perspective on AI and the IoTs in pharmaceutical manufacturing. It is essential to consider how these technologies can enhance transparency and traceability, ultimately strengthening consumer trust in pharmaceutical products and improving supply chain strategies.
– Future Directions and Trends in Technology: AI and the IoTs are continuously changing domains, and research has to be continuously conducted to find emergent trends and future directions. It would be desirable to study the influences that advances like quantum computing or advanced robotics could have on pharmaceutical production with a view to foresight for strategic planning.
– Cross-Industry Comparison: These would also be very informative studies comparing the pharmaceutical industry with industries such as automotive and electronics, where AI and the IoTs have been widely and effectively implemented. These will help in finding out strategies and technologies suitable for transfer to pharmaceutical manufacturing.

The above, if investigated in further research, will go a long way to build up an understanding of the potentiality of AI and the IoTs being used gainfully for innovation and enhancement in pharmaceutical manufacturing.

## 5. Finding and Discussions

Based on the findings of a research study, Table 4 shows some application domains of the IoTs and AI. The content analysis of the articles published between 2019 and 2023 underlines the importance given to agriculture, health, drug manufacturing, and transportation as the most current topics in the specialized literature. Among them, the most extensive studies are being performed on industrial drug manufacturing, which reflects not only its prominence but also its remarkable reliability in practical applications.

**Figure 5:**
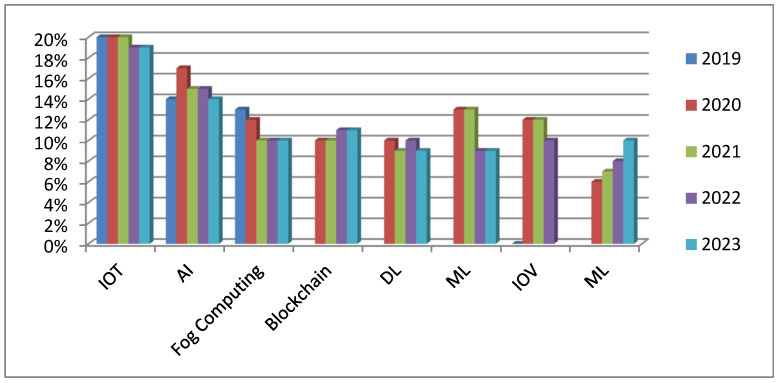
AI and IoTs technologies applications.

Considering the implications of the IoTs and AI in pharmaceutical manufacturing for the period 2019–2023, several technologies play a crucial role, as Figure 5 illustrates. That figure represents the percentage of usage for these technologies such as the IoTs, AI, fog computing, blockchain, edge computing, deep learning, machine learning, and others. If the research in the pharmaceutical sector is analyzed for that period, then the IoTs and AI are found to be the most utilized and focused technologies.

These technological developments are among the major contributors to modernizing and optimizing modern pharmaceutical manufacturing processes for better productivity, safety, and compliance. Integration of the IoTs allows for real-time monitoring of production parameters, quality control, and regulatory compliance, while AI algorithms improve predictive analytics for maintenance and quality assurance, thus enabling reductions in downtime and increased operational reliability.

This has been a period of increased integration of the IoTs and AI in pharmaceutical applications, proving their relevance and contribution to the improvement of productivity and innovation in this industry. Going forward, these technologies are set to play an even more core role in the future of the pharmaceutical landscape by being able to help meet arising challenges and advance manufacturing.

## 6. Quality Control Implications

Changes incorporated into the pharmaceutical industry due to AI and the IoTs are huge in aspects of efficiency, reliability, and reproducibility of drug product manufacture. Both these technologies enable unparalleled levels of operational process automation which can improve quality even to the highest regulatory requirements [105]. Thus, advanced real-time monitoring of each step of the production process is possible and full automation of quality assurance activities becomes possible.

### 6.1. Real-Time Quality Monitoring and Predictive Analytics

The IoTs and AI constitute the steppingstones in migration from conventional reactive kinds of quality control to proactive management. Conventionally, pharmaceuticals depend on periodic quality control based on a human operators’ inference which is based on minute datasets [106]. These allow for monitoring the processes continually with the ability to predict an impending problem so it may be nipped in the bud before the problem snowballs out of control.

AI-driven real-time quality monitoring radically realigns how efficiency, defect mitigation, and refinement in the production process will be realized. Artificial intelligence algorithms for the first time start acting upon a sea of real data, examining minute variations in product quality. According to [107], AI can analyze information emanating from an endless array of sensors on every form and type of machinery or equipment. In other words, this enables the timely detection of abnormalities, reducing waste and minimizing the need for rework. Indeed, these AI systems are much more accurate compared to timely defect detection by a human operator. With predictive capability, AI is enabled to forecast equipment failure through some lessons drawn from the past. Thus, it stands proactive in optimizing machinery operation, reduction in downtime, improvement of overall efficiency, and hence reliability in pharmaceutical manufacturing [108,109].

Studies have highlighted the crucial role of real-time tracking in enhancing quality, supported by data-driven techniques [110,111]. To that end, a CNN reached a rate as high as 99.86% in the visual inspection of manufactured products, proving that AI will enhance the quality manifold in manufacturing.

IoTs-enabled data collection involves embedding sensors in production equipment, continuously gathering critical parameters like temperature, humidity, pH, and pressure. These real-time data become input for AI algorithms, which analyze it for deviations from pre-established quality thresholds. This early detection capability allows manufacturers to address issues before they escalate into serious problems [112,113].

It also predicts possible future quality issues by performing historical-trend analysis and spotting patterns. One can make use of machine learning algorithms by using it to spot fluctuations in the performance of machines or batch quality that are then likely to fail quality tests. This would help the manufacturing sector make proper adjustments in the settings of the machines or their processes, and hence they could avoid very expensive recalls besides reduction in downtime, as recorded by [114].

While the benefits can be felt in AI-driven systems, technology also adds an impressive level of challenges. Success in this domain is highly dependent on high-quality data and algorithms [115]. Certain failures in the precision of any sensor or the failure of correct interpretation by an algorithm may finally result in undiscovered quality problems and hence safety. In addition, the human factor comes into play, and over-relying on automation may easily bypass minor factors that may call for intervention by an expert.

### 6.2. Automated Quality Assurance Systems

Further Enhancing Efficiency and Effectiveness: Among the hallmarks of modern pharmaceutical manufacturing, automation plays a very important role in ensuring product quality at each stage of its production [116]. AI-driven vision inspection can automatically detect small defects that include irregular shapes or dimensions, which could easily escape keen checks from human inspectors. These AI-driven systems provide large-scale quality checks with much higher accuracy compared to manual ones [117,118].

IoTs-Powered Quality Assurance: Real-time monitoring and automation across the facility level are about timely insights into production. Embedding IoTs devices throughout a manufacturing environment allows pharmaceutical manufacturers to realize an unrivaled level of transparency as far as ensuring that in every aspect of production, continuous monitoring is performed and is duly documented [119,120].

Continuous Process Monitoring: IoTs-enabled sensors measure critical process parameters such as temperature, humidity, and chemical composition across continuous modes of manufacture [121]. These processes, like the elaboration of drugs and freeze-drying, are extremely sensitive to the minutest change in the ambient conditions. An IoTs system would monitor all these variables in real-time and send data to a central system for analysis [122]. In case any parameter goes out of the acceptable range, an alarm rings, allowing for immediate intervention before product quality can be compromised.

Closed-Loop Control Systems: In advanced pharmaceutical manufacturing, AI and the IoTs are integrated into closed-loop control systems. These systems automatically adjust process parameters without human intervention. For example, IoTs sensors can detect when the temperature in a tablet compression machine falls outside the optimal range and automatically adjust the temperature or initiate maintenance alerts, preventing the production of substandard products [123,124]. While automated systems enhance efficiency, they also raise concerns about job displacement and the need for skilled operators to oversee these technologies. The initial investment in AI and the IoTs systems can be substantial, posing a barrier for smaller pharmaceutical companies. Moreover, the integration of these technologies requires ongoing maintenance and updates to remain effective, which can strain resources [125].

### 6.3. Maintenance Routines and Troubleshooting Practices

This therefore calls for the need to have manufacturers come up with routine maintenance schedules for AI and the IoTs systems to ensure smooth functionality. The routine maintenance schedule for the systems should include the following [126]:

– Sensor Calibration: Normally, IoTs sensors require frequent calibration so that correct accuracy may be ensured. This mostly includes performance verification of sensors against known standards and performing adjustments where necessary.
– Software Updates: AI algorithms and the IoTs software Versions 23.1 that released in (July-2023) updates leverage new features and enhancements to ensure peak efficiency in the operations of systems.
– Data Management Practices: Strong data management protocols will help with storing, processing, and analyzing data in a very secure and efficient manner. This includes routine backup and verification processes regarding data integrity.

### 6.4. Common Troubleshooting Practices

Alert on Operating Norms: Most of the AI and IoTs systems send out an alert in regard to their operations outside the norm. A review of system alerts gives ways for establishing early problem detection [127].

Diagnostical Tools: Diagnostic tools might be utilized for ascertaining what went wrong with something when malfunctioning occurs. A diagnostics tool studying the data flow or sensor reading spots deviations. For example: DataRobot, Version 7.1. Available from: https://www.datarobot.com/, accessed on 1 January 2025; TensorFlow, Available from: https://www.tensorflow.org/, accessed on 1 January 2025.

Cross-Verification: This will be associated with the assurance that wherever AI systems will give unpredictable output, quality assurance will cross-check their outcomes against actual visual inspections.

Collaborative Problem Solving: It will encourage collaboration among technicians, data scientists, and operators. Troubleshooting can be more effective by combining technical knowledge with practical experience.

### 6.5. Traceability and Documentation: The Key to Compliance and Quality Assurance

Traceability and documentation are at the heart of manufacturing pharmaceuticals, which have a great deal of compliance with regulatory requirements. The integration of AI and the IoTs has been making this tracking capability essentially real-time [128]. IoT sensors integrated with AI systems enable continuous, real-time tracking of raw materials, equipment, and production conditions while maintaining highly accurate records for each batch at every stage of production. This ensures complete traceability, supporting regulatory compliance and facilitating identification of quality issues even after the product has left the factory [129]. For example https://plm.sw.siemens.com/en-US/insights-hub, accessed on 21 February 2025. https://pytorch.org/, accessed on 21 February 2025. https://www.ptc.com/en/products/thingworx, accessed on 21 February 2025.

Although these technologies provide enhanced traceability, they also present challenges related to data security and privacy [130]. The collection of extensive data increases the risk of cyber threats, which could compromise sensitive information. Furthermore, maintaining compliance with regulatory requirements necessitates robust data management practices, which can be complex and resource intensive [131].

### 6.6. Cost Analysis and Economic Benefits of AI and IoTs in Quality Control

Incorporation of AI and the IoTs in the value chain for the manufacture of any kind of pharmaceutical product makes huge impacts on the aspect of quality control both in efficiency and reliability, thus being economically highly beneficial [132].

#### 6.6.1. Economic Benefit

The key economic benefit is the reduction in defect rates. Real-time monitoring using AI brings down the defect rate to almost a minimum. According to certain sources [133], the application of AI in visual inspection can drive accuracy rates as high as 99.86 percent. In this way, the rates of rework and scrap costs are reduced by a great extent. For example, if a facility making USD 10 million annually reduces its rate of defects by just 1 percent, it is bound to manage to save something close to USD 100,000 annually [134].

Other critical reasons exist in the form of gains in efficiency: the IoTs sensors make it easy to allow real adjustments in real-time and for the optimization of temperature, humidity, and pressure among other production parameters. Such proactive monitoring, according to [135], has been able to go as high as 15% in increasing output from production without any attendant rise in the cost of production–cost source. Using the above example, this efficiency could equate to more than an additional USD 750,000 per year to the plant operating at the production cost of USD 5 million.

Advanced technologies contribute further to bringing down the cost of compliance. The pharmaceutical world just happens to be one of the most regulated industries in the world. Advanced AI and IoTs technologies allow for automation of documentation and maintenance of real-time quality records for better compliance without additional audits. Estimates by such a system could determine that companies can save as high as 20% of costs related to compliance which is worth worth hundreds of thousands each year [136].

Predictive maintenance is a contribution to cost-savings and one of the huge areas for economic benefits. Predictive analytics forecast equipment failure by enabling scheduled maintenance. According to [137], companies that apply predictive maintenance can avoid many costs related to unplanned downtime and they can save as much as USD 200,000 per incident [138]. The average cost of unplanned downtime in manufacturing firms is around USD 260,000 per hour, and, therefore, it cannot be that hard to notice the economic benefits.

Last but not least are the lower labor costs due to efficiency. Automation, with the help of AI, reduces most of the time-intensive manual quality checking. According to [139], a firm can save around 30% in the situations of automation relating to the labor-derived cost, and so millions can be saved in just several years because of the quantity of the output that is being produced.

#### 6.6.2. Cost Consideration

While longer-term benefits will be palpable, most of the upfront investments required in AI and IoTs technologies are huge. In one survey, conducted by [140], the average investment for small manufacturers can easily eclipse USD 500,000, inclusive of hardware costs, software licenses, integration support, and user training. The large size of such investment sans solid evidence of certain return on investments can be a big barrier.

Apart from that, these systems require periodic investments for maintenance and upgrades. In the report from Industry Insights, “In 2023”, the updates which are crucial to it devour nearly 10 to 15% of the annual IT budget. That keeps them costing money continuously [141].

Other concerns are regarding the overall cost associated with maintaining this type of information. In particular, large-scale data arising due to the IoTs demands highly resource-intensive systems that enable its manipulation and processing: this can conservatively be thought of at an estimated well in excess of USD 100,000. In addition, costs associated with keeping data compliant with pertinent regulation must also be shelled out [142].

Moreover, one should not forget about human resources and training costs. Connected with the areas that are in need of higher automation, skilled labor becomes in demand. According to [143], average costs to train existing employees or hire new talent in specialized roles could run anywhere from USD 50,000 to USD 100,000 a year—that is a pretty big investment on the part of any manufacturer.

While AI and the IoTs boast improved defect rates, better compliance, and savings through predictive maintenance, the high initial investment keeps operation costs high; proper cost–benefit analysis is required at this point. In other words, the exploitation of all advantages concerning quality control created by AI and the IoTs will require pharmaceutical product manufacturers to weigh immediate expenses against long-term savings and efficiency gains [144].

### 6.7. Examples of Real-Time Monitoring and Automated Systems for Quality Assurance

Indeed, extensive research has been conducted into how well real-time monitoring and automated systems are working in the manufacture of pharmaceutical products for quality assurance. Key among these reasons is the increasing accuracy, reliability, and speed in quality control processes. These new technologies herald a sea change in these traditional QA operations, especially in cases where patient safety and/or regulatory issues feature large in product quality [145].

Consequently, several major pharmaceutical companies have already implemented such systems that guarantee a better quality of manufacturing. A few important examples are identified below:

Temperature and air quality are among the critical parameters continuously monitored in a manufacturing facility of Johnson & Johnson by IoTs-enabled sensors. Real-time data feed-in through sensors, which AI algorithms continually monitor to identify early signs of process deviation. This can enable speedy changes in operations to ensure the quality and consistency of the product.

Boehringer Ingelheim has mounted AI-driven vision inspection systems throughout the production lines. These detect any defects in packaging. It reduces human error and speeds up the inspection of the products; hence, it will be of higher quality since each unit will meet strict regulatory requirements.

Predictive analytics and real-time monitoring are used in vaccine manufacturing at Pfizer. The IoTs-enabled sensors collect data regarding equipment performance and environmental conditions, which are further analyzed by AI models to predict any potential malfunction or deviation. Proactive by nature, Pfizer makes timely adjustments to prevent downtimes and ensures consistent quality in its vaccines.

These companies are just a few examples of how AI and IoTs technologies are inlaid into pharmaceutical manufacturing to make quality certain.

Examples are the integrated vision systems at the stage of pharmaceutical packaging, together with automated systems to make sure every tablet is clear of defects like cracks and size and shape inconsistencies [146]. These are also integrated with feedback loops so that real-time adjustments can be made in the process to rectify the problems pointed out. Real-time monitoring of the environment around an aseptic area gives an effective way to achieve GMP criteria through continuously tracking temperature and air quality [147].

More to this, the IoTs sensors will measure the vital parameters of the process, such as speed and pressure, in order to use AI algorithms with a view to predict the consistency of the mixture during the blending process. Inconsistencies, if there are any, in the system may immediately adjust the process for maintaining uniformity in the product [148].

The use of such advanced technologies helps pharmaceutical manufacturers to control defects to extremely low numbers, and ensure compliance with broad range of regulatory requirement. The revolution will grow to much better levels of greater operatory efficiency, more reliable consistency, and safety for patients with real-time IoTs-driven and AI-based monitoring systems [149].

While successful implementations, such as those by Johnson & Johnson and Pfizer, demonstrate the potential of AI and the IoTs, the scalability of such solutions across the industry varies. Not all companies possess the same level of resources or technical expertise, leading to disparities in the adoption of advanced quality control systems. Furthermore, as these technologies evolve, there is a continuous need for training personnel to adapt to new systems and processes, which can be an ongoing challenge.

## 7. Predictive Maintenance in Pharmaceutical Manufacturing

Pharmaceutical manufacturing is highly dependent on equipment reliability for product quality and a reduction in unplanned downtime. Predictive maintenance, through predictive analytics coupled with real-time data monitoring, offers a more active approach to managing equipment [150]. It helps manufacturers anticipate impending equipment failures by continuously monitoring equipment data to allow corrective action before issues strike an entire production line.

### 7.1. Predictive Analytics of Equipment Failure and Process Deviation

Forecasting predictive maintenance would involve a machine learning algorithm that understands how machinery has historically performed using sensors installed across it. From this understanding, it compiles patterns showing early-wear signs when something is likely to fail. For instance, in real-time, vibration, temperature, and pressure data allow the software to realize an anomaly at the initial stage, such that repairs could be carried out when production is lower [151,152]. Unlike traditional preventive maintenance, where fixed schedules are followed, predictive maintenance will, on the other hand, be dictated by the actual equipment performance data, ensuring that a service is performed only when needed [153].

Anomaly Detection and Forecasting: The algorithms in machine learning learn the way things run normally; therefore, any deviation from normality shows inefficiency or an impending failure. An example is of the pattern of energy use that can depict packaging machinery efficiency losses, where maintenance teams can go on taking remedial measures to avoid costly repairs or the loss of production [154].

**Figure 6:**
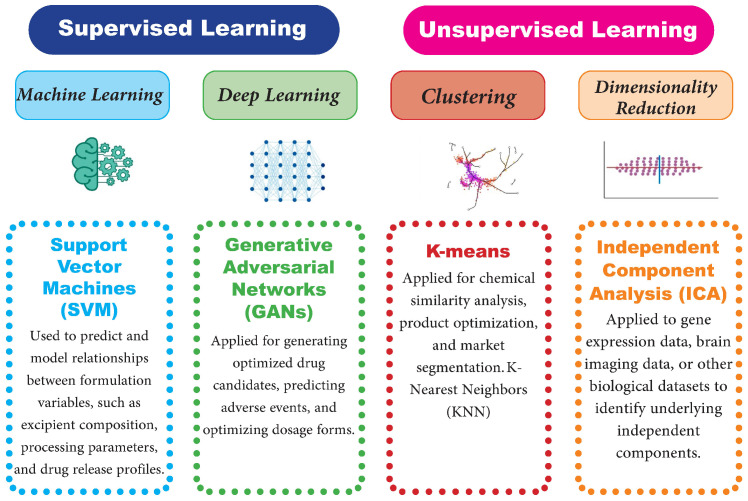
Different AI learning models/tools for predictive pharmaceutical maintenance solutions. For example https://www.tensorflow.org/, accessed on 21 February 2025; https://elevenlabs.io/developers, accessed on 21 February 2025; https://pytorch.org/, accessed on 21 February 2025.

Supervised Learning: These algorithms have very wide applications in predicting equipment failure, using previously labeled historical data. If the historical data reflects when a machine failed and under what conditions (Figure 6), these algorithms can learn to identify patterns leading up to similar situations [155]. Typical ranges of technologies used here can be regression analysis, decision trees, and support vector machines, which enable the forecasting of such estimated outcomes, such as the remaining life of operating equipment or any probability of occurrence of conditions that lead to failures [156].

Unsupervised Learning: The model in unsupervised learning finds the pattern and anomalies in untagged data. In such exploratory data analysis it would be quite useful. Common examples are the uncovering of hidden relationships or behaviors in machine performance data. Clustering categorizes operating conditions that behave similarly or detect atypical instances that may imply impending failures [157].

Supervised and Unsupervised Learning: Most of the predictive maintenance pharmaceutical solutions employ both modes of learning: supervised and unsupervised. To put it differently, unsupervised could be performed in data preprocessing and cleaning and then in clustering, whereas supervised learning is to be performed on certain prediction tasks. As a matter of fact, the integrated approach thus amplifies the accuracy and reliability of the predictive maintenance efforts [158].

### 7.2. Impact of Predictive Maintenance to Reduce Downtime and Maintenance of Production Integrity

The main advantage associated with predictive maintenance is the reduction in unscheduled downtime in industries such as pharmaceuticals, the integrity of production, and timely production. Sudden failure of equipment may bring production to a grinding halt with material wastage and delays of shipments, and it certainly affects not only operational efficiency but also the quality of the final product [159].

Smoothing of Production: Predictive maintenance, by estimating the time a failure is most likely to occur, provides an opportunity for timely intervention in such a way that any repair can be performed well before any failure occurs and hinders the production process [160,161]. Predictive analytics can flag this if it is estimated that a tablet compression machine may suffer a mechanical problem, scheduling the maintenance necessary to replace an apparently worn-out component with a new one, hence preventing its breakdown and enabling the continued running of the production line [162].

Efficiency of Equipment: Predictive maintenance ensures that the equipment is functioning optimally. This would help to reduce waste besides optimizing resources. In pharmaceutical manufacturing, which has to be very precise in its production and efficient in all respects, it assumes much significance. Well-maintained equipment is likely to work within the prescribed limits of operation. Thus, it can help prevent variability that might affect the finished product’s quality [163].

Predictive maintenance will reduce the high costs associated with emergency repairs and expensive component replacements by identifying and rectifying problems well before they create major breakdowns. Besides that, it will also enable the manufacturing industry to optimize their spare parts inventories to maintain only those required and minimize the extra cost of stockpiling [164].

### 7.3. Case Examples of Predictive Maintenance in Pharmaceutical Manufacturing

Various studies have identified that predictive maintenance is able to cause huge improvement in the area of equipment reliability, a reduction in downtime, and optimization in manufacturing processes concerning the pharmaceutical industry. Predictive maintenance allows the manufacturer to estimate the failure of equipment through proper proactive measures in the form of repairs or replacement of such components beforehand, thereby avoiding any occurrence. It is possible due to the inclusion of advanced data analytics, machine learning algorithms, and IoTs sensors [165].

#### 7.3.1. Case Study Evaluations

##### Tablet Production Line

The most documented cases include a leading pharmaceutical company applying predictive maintenance to the manufacturing lines for producing tablets that included very sensitive machinery: tablet presses, encapsulation machines, and coating systems with built-in IoTs sensors monitoring the parameters of vibration, temperature, and pressure—those very indicators reportedly relevant in the literature to an impending mechanical failure [166]. Data were fed, in real time, into machine learning algorithms that would identify patterns of data on historical wear and tears to predict component failures, such as motor bearings or hydraulic pumps.

Outcomes: It reduces unplanned stops in production by 30% because the company can plan maintenance during planned downtime. This approach increases the overall effectiveness of production.

That worked quite well for real-time monitoring and predictive analytics, helping pinpoint failures before they actually happened. Historical data helped in recognizing patterns and thereby gave more accuracy to the predictions.

Some of the things that did not work too well included the initial calibration of sensors and precision in the data collected. This can sometimes result in false positive or missed failures due to the variability in sensor readings, hence the need for robust data management practices.

Improvements in Replication: The training of staff operating the interpretation data will be completed, and scheduling for maintenance will be performed in further implementations. Algorithms will be perfected by incorporating online variations to make predictions pertaining to real-world accuracies better.

##### Filling and Packaging Lines

Another good example could be a multinational pharmaceutical company producing injectable drug products that applied predictive maintenance to high-speed filling and packaging lines, supported by advanced vibration analysis and temperature monitoring for the prediction of motor and pump failures [167].

Output: The predictive maintenance system turned out to be successful in picking up subtle anomalies in machine behavior, thereby allowing component replacements prior to failures, reducing equipment downtime to a great extent, along with averting repairs that would have been very costly.

Anomaly detection in machines early enough to enable timely interventions was so important in maintaining the pace of production. Advanced analytics also allowed the team to find inefficiencies that would have been missed by operators. The anomalies of this system were hardly interpretable at first, a reason that initiated a number of superfluous maintenance activities. It also included huge wastage on scheduled maintenance for which timing was not strategically proper [168].

Improvements for Replication: The model should, again, be developed more in data analytics, maybe adding more diverse data that will further improve the predictive reliability of this model. It would also institute a feedback loop that could enable constant learning from the very maintenance decisions made, thus refining the system in practice over time.

##### Cold Storage Units for Biologics

In that regard, predictive maintenance has been integrated into cold storage units, which are important for tight temperature control in a pharmaceutical company manufacturing high-value and low-volume biologics. The system is able to monitor or predict the failure of refrigeration units through installing temperature sensors [169].

Outcomes: The predictive maintenance model served well in detecting gradual temperature changes, thereby leading to system inefficiencies that may cause deteriorated product quality. It means that the company can, before critical points are reached in the temperature, act to reduce the incidents of product spoilage and loss.

What worked considerably better was how it improved product safety and reduced overall wastage, showing very well how predictive maintenance works in applying a form of insurance to high-value products.

Due to having so many factors that had to be followed simultaneously, an information overload was created in which it was not possible to clearly understand what exactly was wrong with the short-term approach. Additionally, having only one type of sensor limited the depth of analysis.

Improvements for Replication: In refinement, it will be possible for the producer to insert a variety of sensors and more complete data analytics to correlate temperature information with other climatic variables. This would make clear a comprehensive image of storage conditions and make it possible to make necessary rapid interventions if anomalies arise.

##### Challenges and Future Considerations

While the above case studies illustrate the promise of predictive maintenance in pharmaceutical manufacturing, there are a number of challenges. It is important to have high-quality data; incomplete or erroneous data may lead to bad predictions that make operations inefficient [170]. Moreover, scalability is one of the major challenges faced, especially by small manufacturers who might not have all the resources to invest in an advanced predictive maintenance system.

However, these case studies barely touched on such issues of various regulatory environments within which a better understanding of the cost–benefit dynamics is essentially needed mainly with regard to small firms. It is in such contexts of various industry-specific regulations that justify such initial investments, and long-term benefits from the technologies of predictive maintenance should be considered [171].

Predictive maintenance has been a really disruptive approach in managing equipment at the manufacturing level of pharmaceuticals, considering both efficiency and quality demands [172]. Much research is still needed in addressing implementation issues, model development, and scalability over different operational scenarios for the realization of more reliable and safe production processes in pharmaceuticals.

## 8. Regulatory and Compliance Considerations

There is huge potential for realizing efficiencies, improving the quality of products, and making compliance with regulations easier in pharmaceutical manufacturing by using AI and the IoTs. In any case, this industry is one of the most strictly regulated ones. The hard battle for the regulatory bodies remains to evolve their framework to be aligned and address the complexities brought forth in the exponentially growing presence of AI and the IoTs [173]. The section below highlights some of the present landscapes of regulations that have emerged in governance for the aforementioned technologies within pharmaceutical manufacturing and some emerging challenges in sustaining the regimes of compliance and their solutions.

### 8.1. AI and the IoTs in the Pharmaceutical Manufacturing Regulatory Landscape

Among the manufactures with several regulations attached worldwide are pharmaceuticals, guided by various bodies like the U.S. FDA, the European Medicines Agency, and the World Health Organization. But with these technologies of AI and the IoTs gaining momentum, it has been realized by the regulators that the existing policy shall need a facelift lest rigorous oversight be left behind [174].

The FDA has also initiated work on guidelines regarding the way AI and the IoTs can be used at every step of the pharmaceutical process. Within the given framework, the FDA underlined the needs of data integrity and traceability, thus documenting such needs in full, which are the keystones for the transparency of an AI/IoTs system [175]. Their “Guidance for Industry: Data Integrity and Compliance with Drug CGMP” outlined the expectation to have recordkeeping for manufacturing that is both accurate and reliable. That is a pretty basic paradigm shift in AI and the IoTs, improving operational efficiencies but asking for an increasing amount of scrutiny in data management and compliance [176,177].

It also updated guidelines concerning the role of AI and the IoTs in quality control at the level of manufacturing processes. In so acting, it enforces such AI or IoTs to require well-validated mechanisms which should attest to the consistency of performance with an accepted regulatory standard [178]. This does, on the surface, tend to reinforce how the fundamental challenge seeming to underpin this demand appears to be one of how their manufacturing process is going to demonstrate the processes for these systems operating effectively, other than the result in terms of creating auditable data traceable from manufacture into delivery [179].

It affects great bearings based on organizations such as the International Council for Harmonization to set the same standards concerning AI and the IoTs in pharmaceutical manufacturing which offers smoother processes for cross-regulatory platforms, considering that product safety respects international boundaries. The consensus builds itself in relation to a given guide, which also includes automated decision-making and its management.

### 8.2. Emerging Regulations—Challenges in Compliance

While AI and the IoTs hold great promises in pharmaceutical manufacturing, there are major compliance challenges that their introduction into the system raises for manufacturers. Some of the key issues are related to holistic data integrity, system validation, and oversight.

Data Integrity and Traceability: The main regulatory requirements in the manufacture of pharmaceuticals relate to the accuracy, completeness, and reliability of the data from the process [180]. In conclusion, the continuous adoption of AI and IoTs technologies characterizes the continuous collection and processing of huge amounts of data, almost in real-time, originating from different sensors. A simple question it has so far raised for the regulators was to ensure integrity and traceability of data across the production life cycle. Since these IoTs devices create data in the form of temperature and pressure readings, for example, it is the responsibility of the manufacturer to log and justify all changes to that data [181].

This will be a great milestone toward blockchain or encrypted cloud-based integrity of the data (Appendix C). With these solutions, one will have immutable records that are auditable with much ease and allow the manufacturing company to ascertain that the data stays secure and trustworthy [182]. Moreover, AI algorithms will need to be validated periodically by the manufacturers for confirmation of their predictive accuracy and their dependability in decision-making.

Regulation for the Validation of AI and IoTs Systems: This is a call on regulators for the validation of all the systems down the line in manufacturing for constant and expected performance by the authorities, and this poses a challenge in trying to validate AI and the IoTs due to their nature of dynamic changes [183]. These systems will implement learning algorithms that will evolve with time and thus would necessarily have to be continuously validated during their operational life for correctness of functioning, as well as to satisfy regulatory imperatives.

The challenge is huge as it requires the establishment of adequate continuous monitoring and revalidation mechanisms of the AI model for integrating new data into manufacturing entities to sustain high performance and ensure conformance.

Documentation and Audit Trails: All activities in terms of production should be well documented—for example, the manufacture of pharmaceuticals. Coming to the automation of such processes with the help of AI systems, one main challenge that really needs immediate attention is that the documentation has to be traceable, accurate, and complete [184]. These systems will need to create granular audit trails while logging every action AI could take, along with changes in process parameters and equipment settings.

To this effect, the stakeholders in the industry are increasingly engaging with systems that automatically record the tracking. This would make them easily accessible by the regulatory bodies. These can help not only during audits and inspections related to compliance issues but also make them workable regarding a product recall.

### 8.3. Addressing the Challenges of Compliance

Pharmaceutical companies address the compliance challenges posed by advanced technologies in tandem with regulators (Table 5).

#### Some Key Initiatives

AI System Validation and Ongoing Monitoring: There have to be strong processes from an organizational perspective concerning the validation of AI models prior to deploying the same into production [185]. Protocols should address the testing to be performed for precision, reliability, capability for being audited, and traceable outputs, performed by ongoing monitoring of performance and periodic revalidation with an aim at fulfilling the requirements by regulations which remain evolving.

Integrating Blockchain for Data Integrity: Most pharmaceutical firms are now researching ways in which blockchain technology could create tamper-proof records of data generated by both IoTs sensors and AI systems. Blockchain provides a source of truth, a transparent, secure, auditable ledger of manufacturing activities that enhances data integrity while facilitating compliance audits.

Engage Actively with Regulators: It will keep the pharmaceutical manufacturing industry up to date with the dynamic nature of the regulatory environment related to AI and IoTs. This would even extend to setting the boundaries of future rules and standards so that pharmaceutical manufacturers could respond effectively to any change in the regulations with great rapidity [186]. By collaborating with this working group, the FDA is involved in a series of initiatives which are trying to bring clarity and a specification toward a set of actionable guidelines with respect to the application of AI in manufacturing.

Real-Time Data Monitoring and Documentation: Other than ensuring quality right from the continuous manufacturing process, investing in real-time data monitoring systems inherently creates and stores regulatory documentation. The smoothened approach of audit trails provides more significant transparency, making inspections quite a bit easier.

The regulatory landscape is complex; however, it is important to make such complexity a part of modern pharmaceutical manufacturing. That is, the potential of AI and IoTs technologies come into view in case challenges related to data integrity, validation of AI systems, and its documentation will be taken into consideration rightly [187]. Moreover, continuous technological advancements, open dialogue with regulators, and the secure management of critical data help address compliance challenges while enhancing pharmaceutical product quality and adapting to the industry’s evolving expectations.

**Table 5:** Challenges and addresses in traditional pharmaceutical manufacturing as discussed by [188,189].

Challenge	Description	Potential Solutions
Regulatory Compliance	Navigating complex regulatory requirements can delay production and increase costs.	Implementing automated compliance tracking systems and regular audits.
Quality Control	Ensuring consistent product quality can be challenging due to variability in processes.	Utilizing real-time monitoring and AI-driven quality assurance tools.
Supply Chain Inefficiencies	Delays and disruptions in the supply chain can impact production timelines.	Adopting IoTs solutions for real-time supply chain visibility and management.
High Operational Costs	Maintaining equipment and adhering to regulatory standards can be expensive.	Investing in predictive maintenance technologies to reduce downtime and costs.
Data Management	Handling large volumes of data from various sources can lead to errors and inefficiencies.	Implementing integrated data management systems and analytics platforms.
Limited Flexibility	Traditional manufacturing processes may lack adaptability to market changes.	Exploring modular manufacturing systems that allow for quicker adjustments.
Talent Shortages	A shortage of skilled workforce in emerging technologies can hinder progress.	Offering training programs and partnerships with educational institutions.
Environmental Impact	Pharmaceutical manufacturing can generate significant waste and emissions.	Transitioning to green manufacturing practices and waste reduction initiatives.

## 9. Future Trends and Innovations

AI and the IoTs in pharmaceutical manufacturing are in their infancy but hold immense potential to cause transformative changes. As these technologies evolve even further, they will revolutionize production processes, raise product quality, and increase regulatory compliance many folds. This section covers the key developments expected in AI and the IoTs among other emerging technologies likely to further optimize drug manufacturing [190].

### 9.1. Future Developments in AI and the IoTs in Pharmaceutical Manufacturing

Applications of AI and the IoTs in pharmaceutical manufacturing that are current are only indicative of what the future holds. A few such innovations that will drastically change how drugs will be discovered, manufactured, and distributed are discussed below.

AI-based Drug Discovery and Manufacturing: The role of AI in drug discovery is set to expand dramatically beyond its present applications. Now, AI systems analyze big data on the identification of promising candidates, prediction of their efficacy, and elaboration of the optimal protocols for production [191]. In our view, this is an achievement considering the number of studies identifying various limitations that traditional methods face in finding candidates efficiently. Therefore, fully automated AI-driven drug discovery can render the whole process seamless and far less time- and cost-consuming to bring new drugs to market. Others believe that an over-reliance on technologies not supported with human judgment in critical areas is very dangerous.

IoTs Edge Computing in Pharmaceutical Manufacturing: Probably the strongest advantage to integrating IoTs devices into pharmaceutical manufacturing is just there at the edge. In other words, this is the technology in which the processing of information has been initiated right at the edge of the very network. It cuts down on latency to the extreme and therefore increases responsiveness [192]. This is quite critical in key processes, for several studies show even small deviations lead to serious quality problems. We believe that this capability can enable real-time decision-making, but there are also studies showing that successful implementation of edge computing will be required to overcome major integration challenges, especially in legacy systems.

Predictive Analytics for Advanced Drug Manufacturing: While these days AI and the IoTs are being applied to predictive maintenance and quality control, in the future, more predictive analytics will be extended to new areas like supply chain management, demand forecasting, and personalized medicine. A strong split in the research direction surprised me from this perspective: whereas some works underlined advantages created by these technologies to improve manufacturing efficiency, others showed possible risks concerning data security and privacy [193]. Manufacturing by AI and the IoTs in “batch size one” may sound fanciful, but it is going to be the revolutionary future of patient care at the expense of strong frameworks that should guarantee the security of sensitive patient-related data.

Full Automaton in Pharmaceutical Manufacturing: Fully autonomous pharmaceutical manufacturing is no longer a dream. The seamless integration of AI, the IoTs, and robotics has given way to continuous production lines with limited human intervention [191]. Though many researchers shift for efficiency gains after such a seamless integration, we are skeptical about the possible drawbacks this may bring. Workforce displacement and retraining needs that not all the studies consider come along with reliance on automated systems [190].

### 9.2. Future Technologies Under Investigation: Changing the Face of Drug Manufacturing

**Figure 7:**
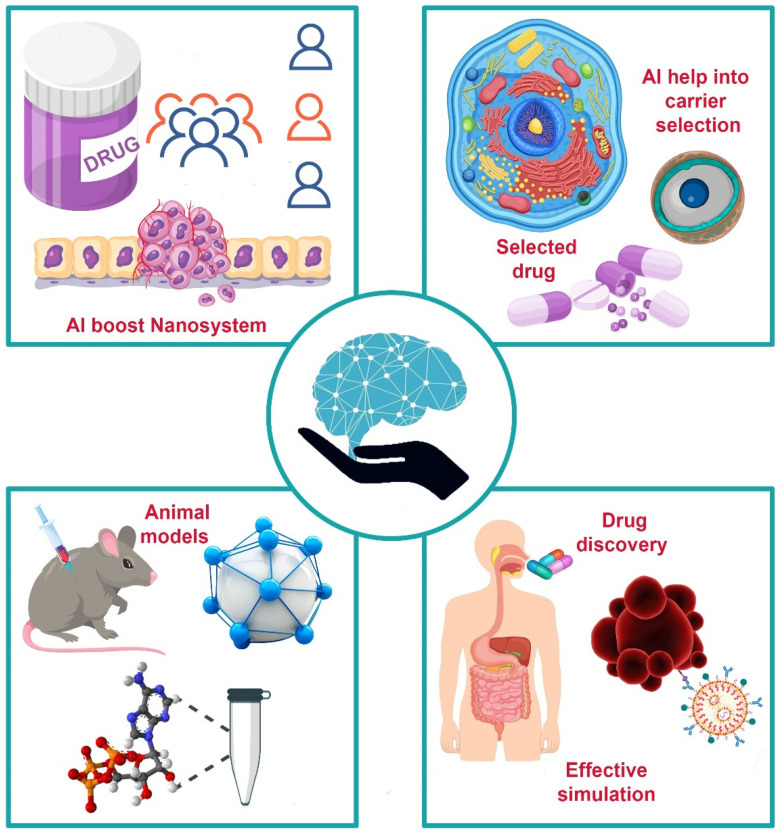
AI contribution to the development of drugs and rise in the precision of selection of parameters and factors in drug design, drug discovery, and drug repurposing methods. It also helps to understand better the mechanism of membrane interaction with the modeled human environment by studying drug permeation, simulation, and human cell targets.

New technologies on the horizon are likely to bring a sea change to pharmaceutical manufacturing (Figure 7), each with promises of efficiency and capability-building.

Blockchain for Data Integrity and Traceability: Blockchain could prove to be something of a game-changer for pharmaceutical manufacturing because it permits secure, indisputable records. Exemplary in this sense is the fact that with clear, tamper-proof records from the sourcing of the very basic level of raw materials up to the final product, blockchain might actually improve regulatory compliance enormously. Some studies support this point, but on the other side, there are opposite ones which mention that blockchain scalability in big supply chains is low, meaning it gives some solutions but still contains challenges in its practicality [194].

Digital Twin Technology in Pharmaceutical Manufacturing: While the very concept of a digital twin, a virtual representation of some physical system or process still barely taking hold within pharmaceutical manufacturing(Appendix D), is still early, there is a general recognition of certain advantages concerning simulation and optimization [195]. Our opinion goes with those studies that raise the bar high by stating the complications in the creation of accurate digital models often requiring substantial time and investment.

Quantum Computing and AI: Though still in the development stage, quantum computing, if coupled with AI, can promise a sea change in the manufacture of drugs. The point at which these technologies meet can solve problems that were earlier beyond classical computing powers. There are thoroughly varied opinions on when practical applications will be possible [196]. Some researchers are overly optimistic; some believe that substantial breakthroughs may be some years away. A balanced view is thus necessary.

3D Printing in Pharmaceutical Manufacturing: The three-dimensional printing technology is among the recent and most innovative approaches so far as personalized medicine manufacturing is concerned. Studies on this subject have presented contrasting perspectives. While some highlight the promising future of on-demand production with fully personalized drugs, others express skepticism, citing significant regulatory hurdles and quality assurance challenges that must be addressed to ensure product safety and efficacy [197].

Wearables and AI in Personalized Medication Manufacture: More wearable devices for continuous health monitoring are a great avenue of real-time data collection. Though many studies trumpet this integration as the transformation that is about to occur in personalized medicine, our view will look at ethical issues around data privacy and comprehensive guidelines that shall manage these data responsibly (Appendix E).

Artificial Intelligence and Robotics in Packaging and Distribution: The merger of robotics with AI will bring in a revolution in packaging and distribution in pharmaceutical manufacturing. We believe that, whereas this reduces error rates and speeds up productivity, at the same time the research community shall take consideration that such automation does affect manpower, including skilled labor personnel who would manage such systems.

### 9.3. Embracing Future Technologies Capable of Further Revolutionizing Drug Manufacturing

Pharmaceutical manufacturing is at that very critical juncture where a host of new technologies converges, including AI and the IoTs. As these powerful tools continue to evolve, their application possibilities will be extended from not only enhancing efficiency but also improving quality, regulatory compliance, and cost reduction. The foreshadowed changes speak to a technological revolution about to take place in the pharmaceutical industry [198].

Such initiatives will see complete success once the pharmaceutical manufacturers show a capability for adapting and integrating evolving technologies quickly [199]. However, my opinion goes to a school of thought that believes in caution and cooperating with the regulatory bodies so that such enhancements, once put into practice, provide factual benefits to the patients and the community as well without making the challenges of the ethics and practicality of living with them tough to face.

## 10. Challenges and Limitations

While the integration of artificial intelligence (AI) and the internet of things (IoTs) in pharmaceutical manufacturing offers significant potential for transforming processes, numerous challenges and limitations must be addressed. These challenges span technical, ethical, operational, and regulatory dimensions, particularly within the stringent framework of the pharmaceutical industry. The following analysis highlights key obstacles, drawing on various studies to illustrate differing perspectives, while also reflecting my own views on how these challenges can be effectively managed [200].

### 10.1. Technical Challenges

Data Integration and Interoperability: One of the most significant technical challenges facing the integration of AI and the IoTs in pharmaceutical manufacturing is achieving effective data integration across diverse systems and devices. Research indicates that most pharmaceutical manufacturing environments utilize a range of equipment, sensors, and software solutions that often lack compatibility. For example, IoTs sensors collect data from various sources, such as temperature and humidity, but integrating this information into a single analytical framework poses significant difficulties. Studies by [201,202] emphasize that enhancing interoperability could substantially improve the efficacy of AI-driven predictive analytics and real-time monitoring. However, these studies often focus on theoretical models without providing practical solutions for implementation. We contend that to realize the potential of these technologies, pharmaceutical companies must invest in standardized protocols that promote seamless data sharing and integration across platforms. This is crucial not only for operational efficiency but also for driving innovation in AI applications, allowing for more comprehensive analyses and informed decision-making.

Data Quality and Consistency: The reliability of AI systems is intrinsically linked to the quality of the data they process. In many legacy manufacturing environments, data collected by IoTs sensors can be inconsistent or incomplete, leading to erroneous predictions and potentially severe operational failures. Various studies, such as those by [203], highlight the importance of high-quality data, yet they often fail to delve deeply into methodologies for improving data integrity. We believe that developing robust data-cleaning techniques and implementing continuous data quality assessments are essential steps in this process. The industry must prioritize these efforts to ensure that AI systems operate effectively as neglecting data quality could compromise product safety and efficacy, ultimately risking patient health.

Complexity of AI Models: The computational demands of advanced AI models, particularly those used for predictive analytics and real-time monitoring, present significant challenges for pharmaceutical manufacturers. Implementing and maintaining these systems requires substantial computational resources and specialized expertise. Several studies, including those by [204], point out the high resource demands placed on companies adopting AI technologies. However, they often underappreciate the logistical and financial constraints faced by many manufacturers. We assert that addressing these complexities through strategic investments in infrastructure and talent development is crucial for the successful deployment of AI technologies. Furthermore, it is vital to create partnerships with tech companies specializing in AI to leverage their expertise in optimizing these systems for the unique challenges of the pharmaceutical industry.

### 10.2. Ethical Challenges

Data Privacy and Security: The ethical implications of adopting AI and the IoTs in pharmaceutical manufacturing are substantial, particularly regarding data privacy and security. The vast amount of data generated include not only product-related information but also sensitive employee and patient data, especially in contexts such as clinical trials. Research by [205] emphasizes the need for compliance with regulations like the General Data Protection Regulation (GDPR) and the Health Insurance Portability and Accountability Act (HIPAA). However, many studies do not adequately address the potential consequences of data breaches and the complexity of implementing robust security measures. We argue that pharmaceutical companies must adopt a proactive approach to data privacy, utilizing advanced encryption methods and strict access controls to mitigate risks. This not only protects sensitive information but also builds trust with patients and regulatory bodies, which is essential for the broader acceptance of AI technologies in the industry.

Bias in AI Algorithms: The effectiveness of AI algorithms is often compromised by biases present in historical training data, which can lead to inaccurate predictions and compromised quality control. Several studies, including those by [206,207], underscore the importance of training AI systems on representative datasets. However, these studies frequently fail to explore the ramifications of biased algorithms on manufacturing outcomes comprehensively. We believe that addressing bias in AI is not merely a technical challenge but a fundamental ethical imperative. Ongoing research into bias reduction techniques must be prioritized to ensure that AI systems operate fairly and effectively. This includes establishing diverse training datasets and regularly auditing algorithms for bias as failure to do so could result in significant quality control failures, jeopardizing patient safety.

Decision-making and Accountability: As AI systems increasingly assume critical decision-making roles within pharmaceutical manufacturing, accountability becomes a pressing concern. The question of liability—who is responsible when an AI system makes a faulty prediction or fails to alert operators to a potential issue—remains contentious. Research by [208] emphasizes the necessity of human oversight in validating AI-driven decisions. However, the ambiguity surrounding accountability frameworks often leaves organizations at a loss regarding how to manage these risks effectively. We contend that developing clear policies for accountability in AI systems is essential to ensure ethical compliance and minimize potential harm. Companies must establish protocols that define the roles of AI and human operators, ensuring that decisions made by AI systems undergo appropriate scrutiny.

### 10.3. Operational Challenges

High Initial Investment and Costs: The integration of AI and IoTs technologies often requires significant initial investments in infrastructure, sensors, and training, which can be prohibitive for small- and medium-sized pharmaceutical companies. Research suggests that many manufacturers hesitate to adopt these technologies without guaranteed returns on investment R&D strategies [209]. This cautious approach can hinder innovation and delay the benefits these technologies could provide. We maintain that while the upfront costs are substantial, the long-term benefits—such as increased operational efficiency, reduced waste, and improved product quality—justify the investment. Furthermore, companies should explore financial models that support gradual adoption, allowing them to scale their investments in line with measurable outcomes and benefits [210].

Scalability of AI and IoTs Solutions: The challenge of scaling AI and IoTs solutions from small setups to more complex manufacturing environments cannot be overstated. Although these technologies may perform well in isolated settings, they often struggle when applied at larger scales, as noted by research from [211]. This scalability challenge can lead to performance degradation and increased operational complexity. We believe that careful planning, along with investment in scalable architectures and modular systems, is essential to ensure that these technologies can meet the increasing demands of larger manufacturing operations. Companies should also consider partnerships with technology providers to facilitate smooth scaling processes without compromising performance.

Integration with Legacy Systems: Many pharmaceutical manufacturing facilities are equipped with legacy systems that were not designed for modern AI and IoTs integrations. This creates significant obstacles to achieving seamless integration, as integrating these older systems with new technologies can be time-consuming and costly. Several studies highlight the challenges associated with compatibility and integration [212]. However, the emphasis on the complexity of these integrations often overshadows potential solutions. We argue that proactive investments in upgrading legacy systems are essential for ensuring seamless integration and maximizing the benefits of AI and IoTs technologies. By prioritizing compatibility, companies can avoid operational disruptions and enhance their overall technological capabilities.

### 10.4. Regulatory and Compliance Challenges

Regulatory Uncertainty: The adoption of AI and the IoTs in pharmaceutical manufacturing is often mired in regulatory uncertainty. Current guidelines from agencies such as the US FDA and EMA provide limited specificity regarding the application of these technologies in manufacturing contexts. Various studies highlight that this ambiguity can slow the adoption of innovative solutions as manufacturers remain skeptical of implementing technologies that lack clear regulatory validation [213]. We believe that engaging with regulatory bodies to develop standardized guidelines is crucial for fostering innovation while ensuring safety and compliance. Such collaboration could expedite the development of clear frameworks that encourage companies to adopt AI and IoTs solutions with confidence.

**Figure 8:**
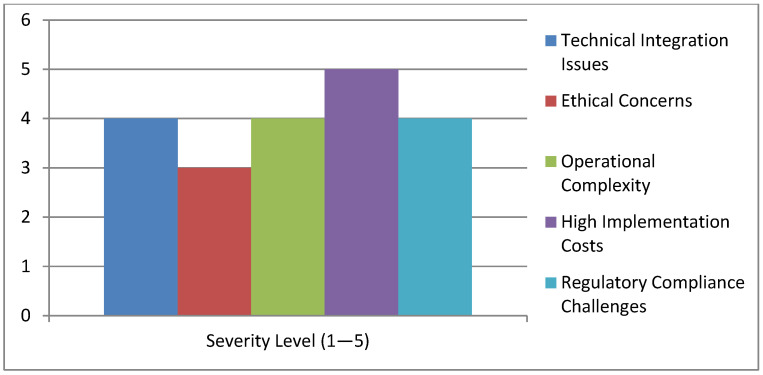
Challenges and limitations of IoTs and AI in pharmaceutical manufacturing.

The integration of the IoTs and AI into pharmaceutical manufacturing has improved considerably between 2019 and 2023, with various bottlenecks and limitations arising on the way. Several of these technologies face challenges for the full realization of their potential and involve technical problems, ethical issues, and regulatory complications. These are represented in Figure 8.

Other operational challenges are further complicating the path of adopting the IoTs and AI. These solutions are very resource-consuming and complex to scale at multiple manufacturing sites. The small firms feel highly challenged by a high implementation cost and limited technical capability that stand in the way of leveraging these transformational technologies in the beginning.

Finally, the ever-changing landscape of regulations adds another layer of intricacy to an ongoing process for manufacturers in trying to keep up to date. While regulations, considering unique IoTs- and AI-related challenges, are continuously being developed, being proactive will help companies avoid sanctions while ensuring safety and efficacy for the devices produced.

This review article critically analyses and discusses the elaborate integration of AI and the IoTs within pharmaceutical manufacturing with regard to improvements and challenges encountered from 2019 to 2023. Critical analysis specifies that quality control, predictive maintenance, and regulatory compliance are merely some of the fields in which the technologies have altered. However, some obstacles remain a cause of concern: data security, ethical problems, and integration into traditional systems.

It advocates that the design of AI algorithms and the IoTs frameworks is such that they cater to the very precise requirements of pharmaceutical manufacturing. For example, there is great relevance in data analytics, which has a significant bearing on the success in case of any real-time monitoring system. This calls for customized implementation of technology against the peculiar requirements of many different manufacturing processes.

The results of the same indicate significant improvements in operational efficiency and regulatory compliance. The paper does indicate various lacunae in the existing literature with respect to the long-term sustainability of these technologies. Further, exogenous factors such as changes in regulations or market scenarios would require greater detail for appropriate strategy implementation.

These challenges notwithstanding, the integration of AI and the IoTs presents unparalleled opportunities to the pharmaceutical industry in terms of raising the level of productivity through increasing its quality. The findings of this chapter are strongly strengthened by the case study and practical examples included that make it useful for industrial players when considering the various complexities of technology adoption [214].

However, the study also points out some limitations in this regard, which might include detailed technical insights that are of interest to specialists. Also, predictions about the future developments of AI and the IoTs are a sensitive issue since such technologies change rapidly, and most predictions may become outdated very soon. Apart from that, it is not to mention a must for advanced technological infrastructure, as such scarcity of the resources for developing area manufacturing regions may hamper the benefits to an extent.

Undoubtedly, this present article will surely contribute much toward the understanding of AI and the IoTs in pharmaceutical manufacturing. It requires more research and discussion ahead with respect to unleashing the complete power of these transformational technologies within the industry due to the emerging challenges.

In conclusion, while the integration of AI and the IoTs in pharmaceutical manufacturing holds transformative potential, a multitude of challenges—technical, ethical, operational, and regulatory—must be addressed. By fostering collaboration among industry stakeholders, technology providers, and regulatory agencies, the pharmaceutical sector can navigate these complexities and unlock the full benefits of AI and IoTs technologies [214]. This collaborative approach will not only enhance operational excellence but also prioritize patient safety, ultimately paving the way for a more innovative and efficient future in pharmaceutical manufacturing.

## 11. Evolving Quality Control Strategies in Pharmaceutical Manufacturing

**Figure 9:**
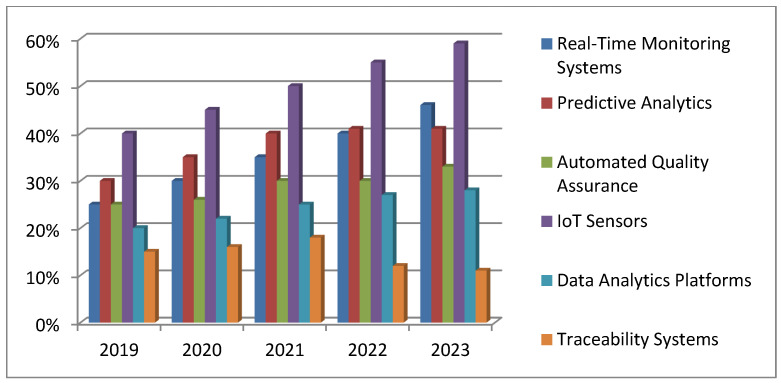
Quality control technologies in pharmaceutical manufacturing.

IoTs and AI technologies have drastically changed the quality control process in pharmaceutical manufacturing from 2019 up to 2023. Many of these technologies, from real-time monitoring systems to predictive analytics and automated quality assurance mechanisms, are at the heart of product integrity and help with compliance issues. Figure 9 below shows the increase in these technologies underpinning the importance of their development in ensuring quality control practices within the industry.

Real-time quality monitoring is also one of the key areas of emphasis, for which research has proven to be true. By allowing the continuous monitoring of critical manufacturing parameters like temperature, humidity, and pressure, manufacturers are in turn allowed to immediately take correct actions that will reduce risks of batch failure and thus keep APIs within specification thresholds. Apart from instilling a culture of quality, integration of sensors with the IoTs enables transparency to operations. This information shall become transparently available for the analytics or decision-making in view [215].

Predictive analytics-driven AI algorithms here go a long way toward computing probable deviations from quality well in advance, on whose basis some genuine preventive measure is brought out from the very end of a manufacturer. ML-based solutions can identify through data whether quality could arise because interventions are very crucial so far as efficiency at large will relate to manufacturing concerns. This has been a period of rising emphasis on the integration of advanced technologies at the level of product quality so that compliance with strict regulatory standards is better achieved.

Besides operational efficiency, the time for regulatory approval was sped up via new processes of quality control. The new product approval time may be reduced with evidence to show how a commitment to quality via robust monitoring and analysis is implemented. This inclusive approach toward quality control turns out not just to achieve compliance but also to enable the viability of the drug manufacturing operation.

### 11.1. Advancements in Predictive Maintenance for Pharmaceutical Manufacturing

Predictive maintenance has also undergone tremendous transformation in the pharma sector between 2019 and 2023 thanks to IoT and AI technologies. As shown through Table 6, some of the technologies employed to achieve this include sophisticated machine learning algorithms, analytics platforms, and even IoT sensors, all of which facilitate high-value decision-making during production [216].

Predictive maintenance, according to studies, is revolutionizing the manner in which companies are managing equipment performance and reliability. IoT sensors have the ability to capture real-time data; now companies will be in a position to anticipate the occurrence of equipment failure, hence tackle it on the basis of need and not time. Aside from minimized downtime, the proactive approach optimizes equipment life with notable cost savings and greater potential for production.

All this in the bigger nudging towards Industry 4.0, where data-driven decision-making and automation more and more are the way forward. The greater need for stable operation has prompted manufacturers to embrace predictive maintenance practices. With the application of IoT-connected sensors installed on high-priority assets, manufacturers monitor performance levels in real-time and are notified with any detected anomalies, thereby allowing interventions to be made in good time [217].

Apart from that, predictive maintenance contributes towards building a culture of continuous improvement within an organization. It is the manner in which producers go about working in their maintenance information, tweaking things along the way to ensure top equipment performance during service. By it, achievement was proved in various case studies: appreciable maintenance expense reduction is accomplished, and uptimes gained from operation. Therefore, IoT and AI might have the potential to be central in transforming the future of pharma manufacturing and effectively meeting the strict regulatory demands.

### 11.2. Policy Support and Government Initiatives

In the context of the USA, policy changes and government initiatives have further bolstered the adoption of IoTs and AI technologies within the pharmaceutical industry. The FDA gave increased support through an initiative on “Pharmaceutical Quality for the 21st Century” and policies related to “Modernization of Manufacturing”, with regard to integrating digital technologies within pharmaceutical manufacturing. These initiatives facilitate a variety of technologies, including IoTs and AI, in surging manufacturing efficiency, ensuring products of the finest quality while paying heed to the regulatory concerns on real-time bases [218].

The creation of funding opportunities by the government in this regard and the establishment of relevant regulatory frameworks have driven innovation in pharmaceutical manufacturing. For example, the FDA’s Case for Quality voluntary initiative aims at advancing advanced technologies in combination with data analytics for advancing drug quality and manufacturing processes [218]. These policies therefore ensure that while the pharmaceutical industry advances with state-of-the-art technologies it does not compromise on its standards of safety and efficiency.

Moreover, the manufacturers in the US are using the provisions in the current American Innovation and Competitiveness Act for the further public–private partnerships necessary to advance such next-generation technology adoption within pharmaceutical manufacturing. In this scenario, the US administration has taken the lead so far in hastening the adaptation of the IoTs and AI for pharmaceutical manufacturing to meet efficiently the upcoming new global challenges that may emerge out of it.

### 11.3. Ensuring Compliance and Regulatory Standards in Pharma Manufacturing

The integration of IoTs and AI technologies into pharmaceutical manufacturing opens up a wide vista of possibilities for better efficiency, productivity, and innovation. However, on the other side, it has indeed raised some real challenges in respect of regulatory and compliance issues. During 2019–2023, the landscape of regulations has seen significant changes with regard to changes in the manufacturing processes themselves attempting to meet emerging standards. Key compliance technologies have come to the fore and include data traceability systems and documentation automation tools. These further ensure that manufacturers remain compliant while embracing new technologies. A detailed overview of these compliance technologies is shown in Table 6.

Adherence to regulatory requirements ranks among the cornerstones of ensuring quality products and patients’ safety. IoTs-enabled traceability and monitoring systems will, therefore, be able to provide real-time compliance checks, hence giving more transparency to the manufacturing chain. Such would include the continuous logging of environmental conditions that will, for one, enable a manufacturer to establish a record of their adherence to GMPs and other set regulatory standards. It helps the manufacturer explain how it meets the regulatory thresholds necessary to ensure the safety and quality of the products [219].

Yet, the challenges remain high in comprehending and standing abreast of, at times, complicated regulatory frameworks, in view of constantly evolving technology at ever-quicker rates. Due to the tremendous development in both the IoTs and AI, even any trivial minute update to the regulations may find its prey among the manufacturers. Precisely, the FDA was deeply concerned with such challenges; thus, from time to time, the department of the FDA issued necessary guidance and updates of rules relating to the application of AI in medicine manufacturing. For example, FDA guidance on “Software as a Medical Device” as recorded by [135] provided detail on how companies could demonstrate that AI-driven systems used in the development and manufacturing of drugs were safe, effective, and compliant. It has also issued guidance on modifications to AI/ML-based software, recognizing that regulatory flexibility will be required to support the ongoing learning and adaptation of AI systems over time.

All this means that pharmaceutical manufacturers will have to develop tracing and documentation of all processes propelled by AI in order to meet these emerging regulatory requirements [220,221]. Consequently, this would call for teamwork in technology deployment: the regulatory affairs and quality assurance and IT departments should collaborate to match regulatory expectations. In addition, to consider any AI application working within the strict standards the FDA sets for safety and efficiency, a great deal of data from the manufacturers would be required to support such reliability, performance, and compliance of the system under review.

Therefore, compliancy in this regard needs to be proactive. While the integration of AI and IoTs technologies may prevail, integrating these from the inception of their coming into existence into regulatory requirements will mitigate the risks and allow new technologies to stay abreast of developing industry standards.

Besides that, the upspring of new technologies needs constant training and education of the staff who participate in matters related to compliance. Awareness among employees, in terms of the implications of the IoTs and AI as far as regulatory processes are concerned, will help not only in being compliant but also contribute towards the culture of accountability. This industry, while embracing these technologies, will have to proactively address the challenges arising in compliance in order to earn confidence from not just the regulatory bodies but all stakeholders.

## 12. Practical Recommendations for Pharmaceutical Companies

Pharmaceutical organizations are effectively using AI and IoTs technologies in their operations based on the following workable recommendations.

– Technology Assessment: Pharmaceutical companies can evaluate the current manufacturing process and clearly identify where exactly AI and the IoTs can bring efficiency, quality, and compliance.
– Pharmaceutical Companies’ Investment and Skill Development: Pharmaceutical companies can invest in the ability to understand new technologies in the right manner and know their operations. Continuous training will help develop innovative and adaptable behaviors. They can partner with technology suppliers for efficient integration, including a support structure, as they are reputable providers within the artificial intelligence- and IoTs-based solutions specific for pharmaceutical manufacturing.
– Pilot Implementation: Very importantly, pilots should be run prior to full-scale implementation that can test the feasibility and effectiveness of applications involving AI and IoTs in controlled environments where changes could be affected based on real data.
– Data Management Focus: Institute good data management practices that ensure quality, security, and compliance of the data maintained; apply analytics in order to arrive at informed decisions and allow for more operational transparency.
– Engage all the regulatory bodies in open communication for more information on compliance issues to ensure the implementation of technology is to the necessary standard.
– Ensure Cybersecurity: A comprehensive cybersecurity strategy will be developed that will protect sensitive data and ensure that data are protected from hacking and cyber threats.

## 13. Conclusions and Future Work

The integration of the IoTs and AI in pharmaceutical manufacturing is transformative in nature and brings huge benefits related to operational efficiency, product quality, and regulatory compliance. A review of literature from 2019 to 2023 shows that applications of IoTs and AI technologies in the pharmaceutical sector, especially within the context of drug manufacturing, have gained tremendous pace, represented by 45 of the 168 articles reviewed. This underlines the increasing trend of dependence on advanced technologies in order to increase productivity and come up with novel ideas regarding manufacturing processes.

IoTs integration therefore allows for real-time monitoring of production parameters for immediate adjustments with a view to optimizing operations. Our results indicate that such integration can reduce equipment downtime by 30–50%, increasing operational reliability many times over. AI algorithms enhance predictive analytics, letting manufacturers predict maintenance needs and decrease operational costs by 15–20% annually. The proactive way not only smooths production but also reduces stops, enabling manufacturing equipment to run at peak capacity.

Moreover, the implementation of AI-powered systems enables quality monitoring without interference at any moment in the production chain. This becomes vital in keeping defective products minimal and in meeting strict regulatory requirements, thus ultimately reinforcing overall safety in pharmaceutical manufacturing. Our study shows that AI and IoTs technologies are the most used, representing about 25% and 19% of the usage rate in drug manufacturing, respectively. These figures show how these technologies are thought of as a gateway to modernization in manufacturing industries.

The future of pharmaceutical manufacturing looks promising, especially when it comes to personalized medicine. That could mean a lot: designing drugs to meet the profile of individual patients, probably with improved therapeutic outcomes, coupled with overall patient satisfaction. Yet, there are challenges in realizing this potential. Key issues include data privacy and security, requiring the development of robust frameworks for data governance that protect patient confidentiality while meeting regulatory requirements.

Furthermore, for the equity of the manufacturing outcome of drugs using AI systems, algorithmic bias within them needs to be addressed. Continued research for identification and mitigation of biases within training datasets shall be key in ensuring fair and effective applications.

The regulatory frameworks also need adaptation for the integration of AI and the IoTs. There should be clear guidelines and criteria by governments that can help in the adoption of such innovations, ensuring GMP compliance, and an environment should be provided which allows the technology to progress. Most pharmaceutical facilities are still on their outdated machinery; therefore, strategies for integrating these new technologies should be cost-effective and compatible with the existing systems for wider adoption. Companies are just starting to scale up AI and IoTs deployments, and as this happens, there will be challenges around computational resources and system complexities. Much research will be required into scalable solutions that assure high performance in diverse manufacturing environments for broader implementation.

It is therefore concluded that while IoTs and AI integration in drug manufacture presents great opportunities for improved operational excellence and better outcomes, some of the challenges presented must yet be overcome through active research and close collaboration between the various stakeholders in the pharmaceutical industry. If it overcomes these barriers by fostering innovative solutions then the full benefits could be realized from these technologies toward the establishment of a more efficient, responsive, and patient-centered manufacturing environment.

## Figures and Tables

**Table 1:** Understanding AI, machine learning, and deep learning.

Term	Definition	Advantages	Disadvantages
Artificial Intelligence (AI)	A field of computer science focused on creating systems that can simulate human cognitive functions, such as learning, reasoning, and problem-solving.	– Can automate complex tasks. – Enhances efficiency in decision-making. – Potential for innovation in various fields.	– Ethical concerns regarding decision-making. – Requires significant computational resources.
Machine Learning (ML)	A subset of AI where algorithms enable machines to learn from data and improve their performance over time.	– Adapts to new data without explicit programming. – Improves accuracy with more data. – Wide range of applications.	– Data quality is crucial; poor data lead to poor results. – May require extensive training data.
Deep Learning (DL)	An advanced branch of ML that uses neural networks to analyze large datasets and identify intricate patterns.	– Highly effective for tasks like image and speech recognition. – Capable of processing unstructured data. – Reduces need for feature engineering.	– Computationally intensive. – Requires large amounts of labeled data. – Often seen as a “black box” with limited interpretability.

**Table 4:** Distribution of articles and the rate of the IoTs and AI usage by application sector (2019–2023).

Sectors Number	Number of Articles	The Rate of Usage of the IoTs and AI in These Domains	
		IoTs (%)	AI (%)
Health	50	30%	35%
Drug manufacturing	45	25%	19%
Agriculture	33	19%	11%
Transportation	24	12%	18%
Smart Cities	16	9%	12%

**Table 6:** Regulatory compliance technologies (2019–2023).

Technology Category	Specific Technologies	Usage Percentage (%)
Data Monitoring	IoTs Sensors, Environmental Monitors	40%
Documentation and Reporting	Electronic Lab Notebooks, Document Control	32%
Compliance Tracking	Blockchain for Traceability, Audit Tools	25%
Risk Management	Predictive Analytics Tools	20%
Training and Education	E-Learning Compliance Modules	15%
Regulatory Intelligence	AI for Regulatory Updates	10%

## Data Availability

No new data were created or analyzed in this study. Data sharing is not applicable to this article.

## Abbreviations

The following abbreviations are used in this manuscript:

IOTS	Internet of Things
ML	Machine Learning
AI	Artificial Intelligence
IT	Information Technology
FDA	Food and Drug Administration

## Appendix A. Data Models and AI Algorithms in Pharmaceutical Manufacturing

This appendix provides an overview of the types of AI models and their applications in pharmaceutical manufacturing.

**Table A1:** AI models used in pharmaceutical manufacturing.

AI Model	Application	Example	AI Model
Supervised Learning	Predictive maintenance, quality control, demand forecasting	Random Forests, Support Vector Machines (SVM)	Supervised Learning
Reinforcement Learning	Real-time process optimization and adaptive control	Process parameter adjustments, adaptive control	Reinforcement Learning
Deep Learning (CNNs)	Drug discovery and formulation analysis	Predicting molecular properties and drug efficacy	Deep Learning (CNNs)
Recurrent Neural Networks (RNNs)	Sequence prediction in drug synthesis or batch production	Predicting drug batch outcomes and process deviations	Recurrent Neural Networks (RNNs)
Generative Adversarial Networks (GANs)	Drug design and molecular structure optimization	Generating novel drug candidates	Generative Adversarial Networks (GANs)

## Appendix B. IoTs Devices and Sensors in Pharmaceutical Manufacturing

This section outlines the role of IoTs devices and sensors in pharmaceutical manufacturing, focusing on their contribution to real-time monitoring.

**Table A2:** IoTs sensors used in pharmaceutical manufacturing.

Sensor Type	Measurement Parameter	Application	Example
Temperature Sensors	Temperature (°C, °F)	Monitor storage conditions for temperature-sensitive drugs	RTD Sensors, Thermocouples
Humidity Sensors	Humidity (%)	Control and monitor humidity levels in clean rooms	Capacitive Humidity Sensors
pH Sensors	pH Level	Monitor pH levels in fermentation or formulation processes	Glass pH Electrodes
Pressure Sensors	Pressure (Bar, psi)	Ensure optimal pressure in manufacturing equipment	Strain Gauge, Piezoelectric Sensors
Vibration Sensors	Vibration (m/s^2^)	Predict machine failure or abnormalities in equipment	Accelerometers, Piezoelectric Sensors

## Appendix C. Blockchain Implementation Models for Pharmaceutical Supply Chain

This appendix focuses on how blockchain can be implemented to ensure traceability and security in pharmaceutical manufacturing and distribution.

**Table A3:** Blockchain models for pharmaceutical manufacturing.

Blockchain Technology	Feature	Application in Pharmaceutical Industry
Public Blockchain	Open, decentralized, transparent	Ensures complete transparency in drug sourcing and distribution
Private Blockchain	Permissioned, centralized, secure	Used within companies to track manufacturing processes and internal data
Hybrid Blockchain	Combination of public and private blockchains	Provides a balanced solution for transparency and privacy within the supply chain
Smart Contracts	Self-executing contracts with predefined rules	Automates compliance verification, shipment tracking, and payment settlements
Permissioned Blockchain	Restricted access, controlled participants	Enables secure tracking of drug production batches, reducing counterfeit risks
Blockchain Technology	Feature	Application in Pharmaceutical Industry
Public Blockchain	Open, decentralized, transparent	Ensures complete transparency in drug sourcing and distribution

## Appendix D. Digital Twin Models in Pharmaceutical Manufacturing

This appendix details digital twin technology, highlighting how it can optimize pharmaceutical manufacturing by simulating production processes.

**Table A4:** Key components of a digital twin in pharmaceutical manufacturing.

Component	Description	Application
Sensors and IoTs Devices	Collect real-time data from equipment and production lines	Continuous monitoring of temperature, pressure, etc.
Simulation Model	Virtual model of the physical manufacturing system	Simulates different production scenarios and forecasts outcomes
Data Analytics Engine	Processes collected data to identify inefficiencies or issues	Identifies production bottlenecks and quality control anomalies
Control Systems	Makes adjustments in real-time based on simulation insights	Adjusts production parameters, optimizing efficiency and quality
Real-Time Feedback Loop	Constant feedback to the physical system to improve performance	Minimizes downtime and maximizes product quality

## Appendix E. Data Privacy and Security Measures in AI and IoTs Integration

AI and IoTs integration in pharma requires robust data protection. Key measures include encryption, access control, and adherence to regulations like GDPR and HIPAA to safeguard sensitive information.

**Table A5:** Key strategies for ensuring data privacy and security in AI and IoTs integration.

Measure	Description	Purpose
Data Encryption	Use of advanced encryption methods (e.g., AES, TLS) to protect data.	Ensures confidentiality and prevents unauthorized access.
Access Control	Implementation of role-based access control (RBAC).	Restricts data access to authorized personnel only.
Anonymization	Anonymizing sensitive patient or production data.	Protects privacy, especially for clinical trial data.
Data Masking	Masking of personal identifiable information (PII).	Safeguards sensitive data in testing and analysis stages.
